# Massive Gene Transfer and Extensive RNA Editing of a Symbiotic Dinoflagellate Plastid Genome

**DOI:** 10.1093/gbe/evu109

**Published:** 2014-05-31

**Authors:** Sutada Mungpakdee, Chuya Shinzato, Takeshi Takeuchi, Takeshi Kawashima, Ryo Koyanagi, Kanako Hisata, Makiko Tanaka, Hiroki Goto, Manabu Fujie, Senjie Lin, Nori Satoh, Eiichi Shoguchi

**Affiliations:** ^1^Marine Genomics Unit, Okinawa Institute of Science and Technology Graduate University, Japan; ^2^DNA Sequencing Section, Okinawa Institute of Science and Technology Graduate University, Japan; ^3^Department of Marine Sciences, University of Connecticut

**Keywords:** RNA editing, plastid-associated genes, dinoflagellate, *Symbiodinium minutum*, hydropathy, light-harvesting complex proteins, minicircles

## Abstract

Genome sequencing of *Symbiodinium minutum* revealed that 95 of 109 plastid-associated genes have been transferred to the nuclear genome and subsequently expanded by gene duplication. Only 14 genes remain in plastids and occur as DNA minicircles. Each minicircle (1.8–3.3 kb) contains one gene and a conserved noncoding region containing putative promoters and RNA-binding sites. Nine types of RNA editing, including a novel G/U type, were discovered in minicircle transcripts but not in genes transferred to the nucleus. In contrast to DNA editing sites in dinoflagellate mitochondria, which tend to be highly conserved across all taxa, editing sites employed in DNA minicircles are highly variable from species to species. Editing is crucial for core photosystem protein function. It restores evolutionarily conserved amino acids and increases peptidyl hydropathy. It also increases protein plasticity necessary to initiate photosystem complex assembly.

## Introduction

Chloroplasts (plastids) are common photosynthetic organelles in eukaryotic algae and land plants. It is generally accepted that plastids first arose when nonphotosynthetic eukaryotic hosts acquired cyanobacterial endosymbionts by a process termed “primary endosymbiosis” (Howe et al. 2008; Keeling 2010). Other nonphotosynthetic eukaryotes may have subsequently acquired endosymbionts from photosynthetic eukaryotes to create secondary plastids (Howe et al. 2008; Keeling 2010). In some lineages (cf. dinoflagellates), secondary plastids were lost and replaced with secondary endosymbiotic plastids or other primary endosymbiotic plastids, resulting in tertiary plastids (Allen et al. 2011). Therefore, the evolutionary history of endosymbiosis and the effects of gene transfer are topics of interest.

Dinoflagellates include both marine and freshwater unicellular eukaryotes belonging to the phylum Alveolata. Approximately 50% of dinoflagellates are autotrophs, whereas the rest are heterotrophs and mixotrophs. They are essential to food chains (Graham and Wilcox 2000). In the crown Phylum Alveolata, containing ciliates, dinoflagellates, and apicomplexans, evolutionary changes in plastid genomes have been dramatic. Ciliates lost plastids and became heterotrophic, whereas parasitic apicomplexans retained an unpigmented plastid remnant termed the apicoplast. On the other hand, two species closely related to apicomplexans, *Chromera velia* and *Vitrella brassicaformis*, are photosynthetic. Their plastid genomes retain ancestral characteristics of both apicomplexan and dinoflagellate plastids and probably share a common red algal endosymbiont (Janouskovec et al. 2010).

Interestingly, rapidly evolving dinoflagellate plastids show a great variety of reduced stages. Their gene content has been dramatically diminished by large-scale transfer of genes to the nucleus, leaving only 12–17 genes in the plastids (Howe et al. 2008). Conventional plastid genomes have all genes physically linked in one molecule, typically 120–200 kb in size (Keeling 2010). In contrast, dinoflagellate plastid genes reside on small plasmids of 2.2–6 kb, termed “minicircles” (Zhang et al. 1999), containing a few genes and a core, noncoding region, which is conserved within species and plays a regulatory role (Zhang et al. 2002; Leung and Wong 2009; Wisecaver and Hackett 2011). Moreover, a number of unusual postranscriptional RNA modifications, including the addition of 3′-terminal poly(U)tracts, occur in the ancestral chloroplasts of dinoflagellates. Extensive RNA editing occurs in some dinoflagellates (Zauner et al. 2004; Wang and Morse 2006; Dang and Green 2009), employing diverse editing types that have not been observed in mammals and plants. This leads to a speculation about the functional interconnection between poly(U)tailing and RNA editing in dinoflagellates plastid transcripts (Dang and Green 2009).

We chose to study *Symbiodinium* because it is a symbiotic, photosynthetic partner of corals, and coral bleaching epidemics involve responses of *Symbiodinium* plastids to global environmental change (Takahashi et al. 2008). Some species of *Symbiodinium* can be cultured in the laboratory, facilitating experimentation. We characterized three categories of plastid-associated genes from *Symbiodinium minutum*: 1) Plastid-encoded genes encoded in DNA minicircles of the plastid genome; 2) plastid-transferred genes that were probably plastid-encoded originally but subsequently were transferred to the nuclear genome; and 3) nuclear-transferred genes that were probably acquired directly from the nucleus of the previous plastid host (Bachvaroff et al. 2004). To better understand dinoflagellate evolution and the results of gene transfer from endosymbiotic algae, we focused on plastid-encoded and plastid-transferred genes. We examined only those nuclear-transferred genes that encode subunits of the photosynthetic apparatus (Allen et al. 2011). RNA editing types and possible consequences of plastid-encoded gene editing in *S. minutum* were also revealed in this study.

## Materials and Methods

### Sequencing, Assembly, and Mapping

Plastid-associated gene sequences of *S. minutum* were obtained from whole-genome sequencing using next-generation sequencers (Shoguchi et al. 2013). The assembled genome, gene model ver. 1.2, and transcriptome contigs are publicly available via a genome browser (Koyanagi et al. 2013). In our previous study (Shoguchi et al. 2013), construction of a transcription start site (TSS) library and sequencing was performed as described in Yamashita et al. (2011). Cells for TSSs analysis were cultured at 25 °C in a 12 h light/dark cycle and collected during a light cycle. Briefly, total RNA was treated with 2.5 U of BAP (TaKaRa) at 37 °C for 1 h and 40 U of TAP (Ambion) at 37 °C for 1 h. Then BAP-TAP-treated RNAs were ligated with the RNA oligonucleotide. After DNAse treatment, poly(A)-containing RNA was selected and subsequently used for library construction. Sequencing reactions were performed according to Illumina’s instructions for the Hiseq. Mapping of TSS reads and transcriptome reads was performed using Bowtie (Langmead et al. 2009) with default parameters. Enriched positions of the 5′-end of mapped reads (>50) were defined as candidates for TSS.

### Molecular Cloning to Verify Plastid Minicircles

Each *S. minutum* DNA minicircle contained conserved, noncoding sequences (CNSs) of about 600–1,000 bp with both highly conserved and variable regions (V regions). Software assemblers were confused by repetitive sequences and could not reliably manage them. Therefore, to identify these genes, we performed molecular cloning using inward and outward-directed polymerase chain reaction (PCR) amplification and subsequent sequencing of minicircle plastid genes. PCR conditions for all amplifications were 95 °C for 3 min; then 15 cycles of 94 °C for 20 s, 68 °C for 2 min; then 25 cycles of 94 °C for 20 s, 57 °C for 20 s, 68 °C for 1.5 min; followed by 68 °C for 5 min, using a Bio-Rad DNA Engine Tetrad2 Peltier Thermal Cycler. Reagents in each 50 μl reaction were 20 pmol each primer, 100 ng genomic DNA, 0.25 μl of Biotaq (Bioline), 0.1 mM (final concentration) each, dATP, dCTP, dTTP, and dGTP, 5 μl of 10 × PCR buffer, 120 mM MgCl_2__,_ and ultrapure distilled water. Amplicons with the expected sizes were purified using a gel extraction kit (QIAGEN). These were directly sequenced with each of the amplification primers using an ABI 3130xl DNA Analyzer.

### RNA Editing Detection

First, it should be noted that DNA and RNA sequence data of *S. minutum* were generated from haploid clones, therefore mismapping due to single-nucleotide polymorphism (SNP) is unlikely. Plastid gene sequences were from PCR cloning and an ABI sequencer; therefore, sequence information is independent of short read assembly. Because organelle genomes have high copy numbers in the cell, high-quality DNA-seq reads provided more than 300-fold coverage using Bowtie. Mapping of DNA-seq reads to plastid ABI sequences using BWA default settings (Li and Durbin 2010) showed low genetic variation of plastid genes, 0–0.01% of SNP. To identify RNA editing sites of plastid genes, RNA-seq reads were mapped to genome and transcriptome contigs using Tophat (Trapnell et al. 2009) with default settings, which allow two mismatches per read map. Our method was slightly different from the strategy used in human RNA editing identification (Lin et al. 2012), but the possibility of technical artifacts as described in Pickrell et al. (2012) was evaluated from the same point of view. Because the RNA editing rate in organelle genomes of dinoflagellates is high, the number of mismatches per read was high, resulting in a low number of RNA-seq reads that mapped specifically to plastid genes. Therefore, to get more specific reads, we retrieved approximately 1.2 million RNA-seq reads that mapped to plastid transcriptome contigs, removed duplicates, and then mapped back to DNA sequences using BWA. Moreover, to ensure that no variation in the *S. minutum* genome confounded our results, SNPs detected among DNA-seq reads by SAMtools (Li et al. 2009) were removed. To avoid false-positive RNA–DNA differences (RDDs), in which all reads align only in one direction and mismatch sites appear at the end of the alignment, each variant alignment was checked manually. Edited sites with more than 10-read coverage and no strand or position bias were called.

### Prediction of Three-Dimensional Structure

Homology modeling using the automated mode of SWISS-MODEL (http://swissmodel.expasy.org/, last accessed June 16, 2014) was performed (Kiefer et al. 2009). The resulting three-dimensional (3D) structure prediction (Protein Data Bank file) was described using the molecular graphic software, Waals 2013 (Altif Laboratories Inc.). Because template proteins for psaB, psbC, and psbE are not predicted properly using the SWISS-MODEL database, MATRAS (http://strcomp.protein.osaka-u.ac.jp/matras/, last accessed June 16, 2014) (Kawabata 2003) was used to search the PDB for a suitable template protein. Secondary structures were assigned on Waals 2013, using dictionary of protein secondary structure (Kabsch and Sander 1983).

### Comparison between Predicted Proteins Before and After RNA Editing

The 3D structures are obtained using predicted proteins from mRNAs without RNA editing. The intervening sites with stop codons in petB and petD are substituted to 136 S and 32 W, respectively, by RNA editing. Predicted proteins before and after RNA editing are superimposed using the auto-fit function of Waals, which finds common structures for maximum number of superimposed amino acid and minimum root-mean-square distance (RMSD). Less than a 3 -Å cut-off distance between superimposed structures indicated no significant conformational change.

### Data Analysis Software

To identify plastid-associated genes, BLASTP (Altschul et al. 1990) and Pfam domain searches (Finn et al. 2010) were performed using plant and algal plastid-encoded genes as queries. Red and green algal plastid-encoded genes and nuclear-transferred genes encoding the photosynthetic apparatus were also included in this analysis. In addition, KEGG annotation (Moriya et al. 2007) was applied to most *S. minutum* genes to determine their functions. Alignment of protein sequences was done using MAFFT (Katoh et al. 2002) and ClustalW (Larkin et al. 2007). Estimation of molecular weight (MW) and grand average of hydropathy (GRAVY) scores of plastid genes was accomplished with ProtParam (Gasteiger et al. 2003). tRNAscan-SE 1.21 (Schattner et al. 2005) was used to search for tRNA in plastid minicircles. Analysis of candidate promoters was performed using the Neural Network for Promoter Prediction (NNPP) version 2.2 (Berkeley Drosophila Genome Project, http://www.fruitfly.org/index.html, last accessed June 16, 2014).

## Results

### Plastid-Associated Gene Repertoire in *S. minutum*

**Fig. 1.—:**
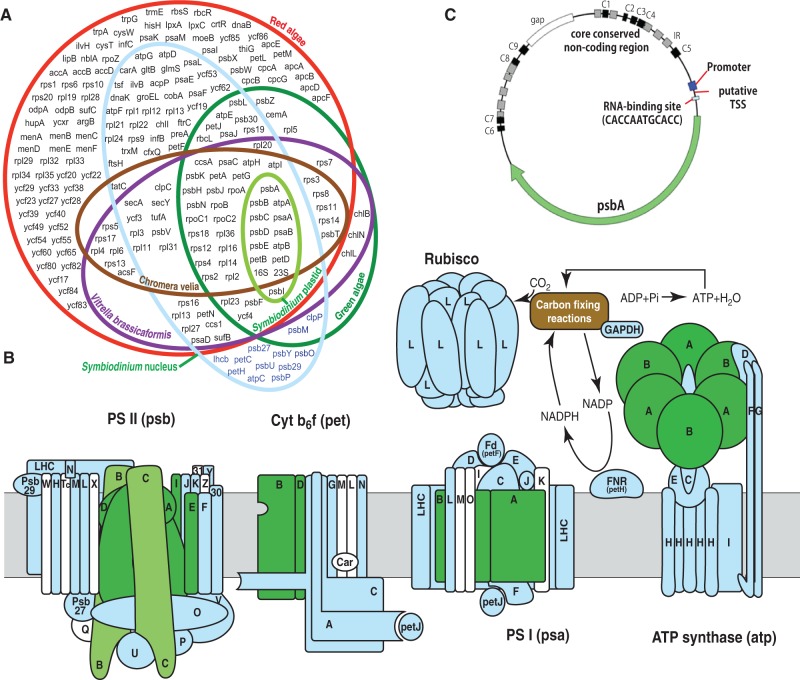
*Symbiodinium minutum* plastid-associated gene content. (*A*) A Venn diagram of plastid-associated genes in the plastid genome (green) and the nuclear genome (blue). Gene content of chloroplast genomes of closely related apicomplexans, *Chromera velia* (Janouskovec et al. 2010) (brown) and *Vitrella brassicaformis* (purple) (Janouskovec et al. 2010; Obornik et al. 2012), the red alga, *Cyanidioschyzon merolae* (Ohta et al. 2003) (red), and the green alga, *Chlamydomonas reinhardtii* (Maul et al. 2002) (dark green) are also shown. (*B*) Major proteins and protein complexes of the plastid photosynthetic apparatus. Polypeptide subunits of plastid-transferred genes are colored green; nuclear-transferred genes are colored blue, and those not found in the *Symbiodinium* genome are white. At least in PS I, PS II, and the Cyt b6/f assembly, plastid-encoded subunits are required for initiation of complex assembly, which is a highly ordered process (Nickelsen and Rengstl 2013). PsbE, PsbI, PsbA, and PsbD subunits are first transiently accumulated to build a subcomplex, and then PsbB and PsbC (light green) are incorporated later, for assembly of PS II (Nickelsen and Rengstl 2013). Assembly of PS I is initiated by membrane insertion of PsaA and PsaB, forming a heterodimer, which accounts for almost half the molecular mass of the mature complex (Schottler et al. 2011). In the Cyt b6/f complex, first Cyt *b*_6_ (encoded by *petB*) and PetD form a subcomplex that serves as a template for assembly of Cyt *f* (encoded by *petA*) and PetG (Wollman 1998). (*C*) *psbA* minicircle of *S. minutum.* The core-conserved region contains a high density of IRs (gray) and nine conserved regions (black). Another conserved region shown between C5 and a coding region is a candidate promoter (blue) located just before the TSS. The “CACCAATGCACC” motif (light blue) found in all minicircles containing protein-coding genes is a putative RNA-binding site. A “GAP box” in white represents incomplete sequences that were estimated from PCR products. Compared with red algae (red), the number of plastid-associated genes has been greatly reduced in *Symbiodinium*. However, in *Symbiodinium,* the overwhelming majority of these are located in the nucleus (pale blue), rather than in plastids (light green).

Using genome-wide analysis, we first characterized plastid-associated genes in the *S. minutum* genome, the first decoded photosynthetic alveolate genome (Shoguchi et al. 2013). Only 14 of 109 plastid-associated genes are plastid encoded. These are present as DNA minicircles in the plastids, whereas the remaining 95 genes with spliceosomal introns are found in the nucleus (fig. 1 and supplementary table S1, Supplementary Material online). Rare intronless genes, such as *psbH*, are mapped onto scaffolds that have other genes with introns (supplementary table S1, Supplementary Material online). Among 95 genes, 83 are probably plastid-transferred genes, because they occur in the plastid genome of red algae (Ohta et al. 2003), and 12 genes (fig. 1) are nuclear-transferred genes, as they are encoded in the rhodophyte nuclear genome. Interestingly, many plastid-transferred genes had two or more gene models showing the presence of duplicated genes, which are more than 80% identical but very diverged from their sister group (apicomplexans) and from red algae (supplementary table S1 and fig. S1, Supplementary Material online). For example, *psbH* had six models (*psbH1–psbH6; psbH3–6* were present on one scaffold) (supplementary fig. S1, Supplementary Material online), *psbL* and *psbM* had four models each (*psbL1–psbL4* and *psbM1–psbM4,* respectively).

The nuclear-transferred gene encoding light-harvesting complex (LHC) proteins was the most heavily duplicated. There were 101copies of *lhcb* (supplementary table S1, Supplementary Material online). Meanwhile in other dinoflagellates, only 10–23 genes have been reported, either by Bachvaroff et al. (2004) or in the NCBI EST database. This is a much higher number than reported in other dinoflagellates. In addition, half of them are expressed as polyproteins containing multiple LHC polypeptides (supplementary fig. S2, Supplementary Material online), as reported in other dinoflagellates (Hiller et al. 1995). In dinoflagellates, LHCs bind to chlorophylls (Chl) *a* and *c* and to carotenoids; therefore, sometimes they have been called Chl *a/c* binding proteins. Hoffman et al. (2011), defined seven Chl *a/c-*binding protein subfamilies based on phylogenetic tree reconstruction. Using that work as a reference, our phylogenetic tree analysis (supplementary fig. S2, Supplementary Material online) showed that gene expansion has occurred mainly subfamily VII, particularly subfamilies VIIa and VIId (Hoffman et al. 2011), which are specifically in peridinin dinoflagellates. Therefore, it is likely that gene expansion in this family occurred mainly after the divergence from a dinoflagellate common ancestor. Expansion has also occurred in subfamily IIIa1(Hoffman et al. 2011), which groups sequences from dinoflagellates, diatoms, and haptophytes. Many plastid-associated genes in *S. minutum* were duplicated after transfer into the nucleus, perhaps with the function of ensuring photosynthesis in the reduced incident light due to the symbiotic life style of *Symbiodinium*.

### Comparison of Gene Content

*Symbiodinium* plastid-associated genes were compared with those of the green alga, *Chlamydomonas reinhardtii* (Maul et al. 2002), the red alga, *Cyanidioschyzon merolae* (Ohta et al. 2003), and two apicomplexans, *C. velia* and *V. brassicaformis* (Janouskovec et al. 2010). Thirty-five genes were common among them. The *Symbiodinium* plastid genome was the smallest (fig. 1). Thus, *psbI* in the *Chromera* plastid genome was independently lost by *Symbiodinium* or transferred to the nuclear genome. Comparisons with the *Cyanidioschyzon*, *Chromera*, and *Vitrella* plastid genomes indicate that since the divergence from the alveolate common ancestor, at least 15 plastid-associated genes have been lost or diverged in *Symbiodinium*. These are mainly ribosomal proteins, which may be complemented by nuclear ribosomal proteins, especially because only 12 protein-coding genes have been retained in the plastid. The majority of *Symbiodinium* plastid-associated genes (43 genes) were transferred to its nuclear genome, leaving only 14 genes in plastid DNA minicircles. Gene transfer from plastids to the nucleus probably occurred since the last common ancestor of dinoflagellates and apicomplexans, although the possibility of independent transfers in each lineage after splitting cannot be excluded. We hypothesize that gene transfer to the host nuclear genome occurred after endosymbiosis of red algae by the alveolate ancestor and that this process occurred several times.

*Vitrella* and *Symbiodinium* possess 12 plastid-associated genes not found in *Chromera*. These may have been lost independently or transferred to the *Chromera* nuclear genome. In terms of plastid gene content, more genes are shared between the plastid genomes of *Symbiodinium* and *Vitrella*, than with *Chromera*. To understand their relationship, molecular phylogenetics based on maximum-likelihood (ML) analysis was carried out using plastid-encoded genes shared with *Symbiodinium*. ML trees (supplementary fig. S3, Supplementary Material online) gave a result consistent with findings of Janouskovec et al. (2010), suggesting monophyly of alveolate plastids where the alveolate clade (including *Symbiodinium*) and the Stramenopile clade are more closely related to the Haptophytes/Crytophytes, than to red algae. These results support an evolutionary scenario in which symbiosis of red algae with the common ancestor of Chromalveolates (Alveolata, Stramenopiles, Haptophyta, and Cryptophyta) resulted in a secondary plastid complement among dinoflagellates. *Symbiodinium* is closely related to *Chromera* and *Vitrella*; however, their plastid genes have evolved very rapidly, as shown by a long branch in the phylogenetic tree (supplementary fig. S3, Supplementary Material online). Their plastid genomes may have coevolved with extensive structural modification, being reconfigured into minicircles, as shown for other dinoflagellate species (Zhang et al. 2002).

### Structure of DNA Minicircles

Combining data from PCR sequencing and genome assembly, DNA from 14 *Symbiodinium* minicircles was characterized (fig. 1). The size of these minicircles ranged from 1.8 kb (*psbE*) to 3.3 kb (*psaA*) (table 1). In contrast to previous dinoflagellate studies that found two or more plastid genes per minicircle (Hiller 2001; Nisbet et al. 2004; Barbrook et al. 2006; Dang and Green 2009), in *Symbiodinium*, only a single plastid gene was present in each minicircle. No empty minicircles (lacking protein coding genes) or minicircles containing tRNA were found. No genes had introns or predicted signal peptides, but they did display a core containing CNSs (fig. 1*C* and supplementary fig. S1, Supplementary Material online). The highly conserved CNSs could be easily aligned, although some insertions and deletions were evident (supplementary fig. S4, Supplementary Material online). However, CNSs were species specific. Consistent with reports from other dinoflagellates (Zhang et al. 1999; Howe et al. 2003), they could not be aligned with sequences from other *Symbiodinium* species of clade C (Moore et al. 2003), even though they share some features, such as a high density of GC-rich inverted repeats (IR).

**Table 1:** RNA Editing Types in 14 Plastid-Encoded Genes of *Symbiodinium minutum*

Gene	Transcriptome ID	Estimated Size of Minicircle (kb)	No. of Edits (%)^a^	Editing Type									No. of Amino Acid Substitutions (%)^b^	gDNA/cDNA Identity to *Heterocapsa triquetra*
				A/G	G/A	C/U	U/C	G/C	G/U	U/G	A/C	A/U		
*psbA*	symbB1.comp0_c0_seq1	2.4	4/1,029 (0.4)	2	1	0	0	1	0	0	0	0	3/343 (0.9)	86/86
*psbB*	symbB1.comp28_c0_seq1	2.5	30/1,500 (2.0)	18	3	4	5	0	0	0	0	0	28/500 (5.6)	68/72
*psbC*	symbB1.comp52_c0_seq1	2.5	25/1,359 (2.4)	15	0	4	2	3	0	0	1	0	22/453 (4.9)	73/76
*psbD*	symbB1.comp12_c0_seq1	2.25	8/1,074 (0.7)	3	0	0	3	1	0	1	0	0	7/358 (2.0)	88/88
*psbE*	symbB1.comp2_c0_seq1	1.8	9/234 (3.8)	4	0	1	0	2	1	0	1	0	8/78 (10.3)	67/71
*psbI*	symbB1.comp1832_c0_seq1	2.13	3/108 (2.8)	0	0	1	1	0	1	0	0	0	3/36 (8.3)	45/45
*petB*^c^	symbB1.comp54_c0_seq1	2.3	23/657 (3.5)	5	1	2	6	9	0	0	0	0	22/219 (10.0)	67/72
*petD*^c^	symbB1.comp26_c0_seq1	2.2	33/477 (6.9)	12	4	2	6	8	0	0	1	0	28/159 (17.6)	51/54
*psaA*	symbB1.comp56_c0_seq1	3.3	100/2,022 (4.9)	52	8	20	13	2	0	0	5	0	84/674 (12.5)	56/60
*psaB*	symbB1.comp37_c0_seq1	3.2	85/2,103 (4.0)	53	5	15	6	4	0	0	1	1	78/701 (11.1)	54/57
*atpA*	symbB1.comp193_c0_seq2	2.4	43/1,434 (3.0)	28	3	5	6	1	0	0	0	0	37/478 (7.7)	67/70
*atpB*	symbB1.comp144_c0_seq1	3	50/1,971 (2.5)	29	3	5	10	2	0	0	1	0	44/657 (6.7)	51/53
*16 S rRNA*	symbB1.comp6517_c0_seq1	2.35	22/794 (2.8)	17	0	15	0	0	0	0	0	0		
*23 S rRNA*	symbB1.comp210_c1_seq10	2.5	36/1,138 (3.2)	26	0	7	0	1	2	0	0	0		

Note.—By comparing with other dinoflagellate genes, three alternative start codons were predicted: UUG (prokaryote start codon) for *psbI*, AUA (possible start codon of *Heterocapsa triquetra* plastid gene) for *petD*, and UUU for *psaB*.

^a^Edits in coding sequences were counted.

^b^Including a signal from stop codon.

^c^One of the editings in each gene convert a conventional stop codon to a sense codon, translating into 136th Ser of petB and 32nd Try of petD.

In *S. minutum,* CNSs had a high density (15–20) of IR. At least nine conserved CNS IR regions (C1–C9) were found, embedded in a V region of incomplete sequences (fig. 1*C*). Most C regions had a high GC content, implying that stable hairpin structures may be formed. V regions contain variable numbers of IRs, and they often have a high AT content (supplementary fig. S4, Supplementary Material online). Directed repeats, which are more common in CNSs of *Heterocapsa,* were found in V regions of *Symbiodinium petD* and *psbE* (supplementary fig. S1, Supplementary Material online). All minicircle genes displayed the same orientation with respect to CNS, as shown in other dinoflagellates (Zhang et al. 1999). These common features of *S. minutum* minicircle CNS suggest that it functions as the origin of replication (Zhang et al. 2002).

**Fig. 2.—:**
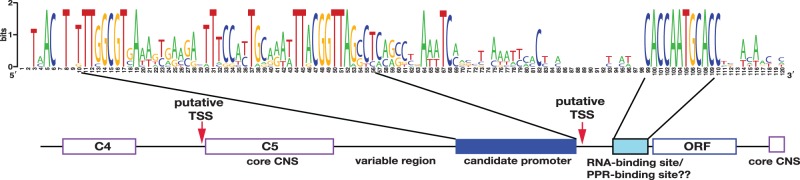
Model for transcription of plastid minicircles. Polymeric and monomeric transcripts, longer than a minicircle, are transcribed via a rolling circle mechanism and require further processing (Dang and Green 2010). Major transcripts start after a prokaryote-type promoter, shown in the sequence logo. Another possible start site was suggested from TSS analysis. The conserved motif found downstream of the TSS is a putative RNA-binding site, which presumably interacts with PPR proteins and blocks 5′–3′-exonuclease trimming. The length of the V region between the TSS and the RNA binding site is 1–41 bp in length.

So far, the 5′-structure and the transcription start site (TSS) have not been identified in dinoflagellate minicircles; however, integrative transcriptome analysis, which recognizes the cap structure of mature mRNA using “Oligo-capping,” suggested that most putative TSSs are between C5 and the ORF of plastid-encoded genes (fig. 2 and supplementary fig. S5, Supplementary Material online). Because the TSS library was constructed for nuclear transcripts, poly(A)-containing mRNAs were enriched; however, organelle transcripts containing modifications also appeared because of the high expression level. As described in the Materials and Methods section, this library will identify both primary and processed transcripts; therefore, many TSS reads mapped to the ORF are from processed transcripts and were removed. Because of technical difficulties, an appropriate negative control excluding processed transcripts cannot be made. However, an independent cDNA library without BAP and TAP enzyme treatment showed that more than 5,000 reads consistently cover the ORF region but not the 5′-UTR of plastid genes. Because a significant number of reads from the TSS library cover the 5′-UTR (supplementary fig. S5, Supplementary Material online), this suggests that TSS library was selective for the TSS. Alignment of the TSS regions of 12 minicircles that contained protein-coding genes clarified two conserved sequences (upstream and downstream of the putative TSS) (fig. 2). The upstream sequence did not show the typical canonical promoter; however, promoter prediction with NNPP version2 suggested that it contains a prokaryotic promoter. This implies that minicircle genes are transcribed by a eubacterial-type RNA polymerase, encoded by plastid-transferred genes, *rpoA*, *rpoB*, *rpoC1,* and *rpoC2*.

Aberrant minicircles resulting from DNA rearrangements have been reported in many dinoflagellates (Zhang et al. 2001). In *Symbiodinium*, aberrant minicircles composed of CNS and large deletions in the coding region of *atpA, atpB, psaA, psbB,* and *psbD* (supplementary fig. S1, Supplementary Material online). It is likely that these minicircles were formed by replication slippage. Recombination may occur infrequently, because we have consistently found only one gene per minicircle. RNA-seq data showed no support for these truncated forms, suggesting that mature RNA were not generated; thus some selective replication advantages, or a lack of negative selective pressure, may promote retention of these aberrant minicircles in the genome.

### RNA Editing in the Plastid Genome

**Fig. 3.—:**
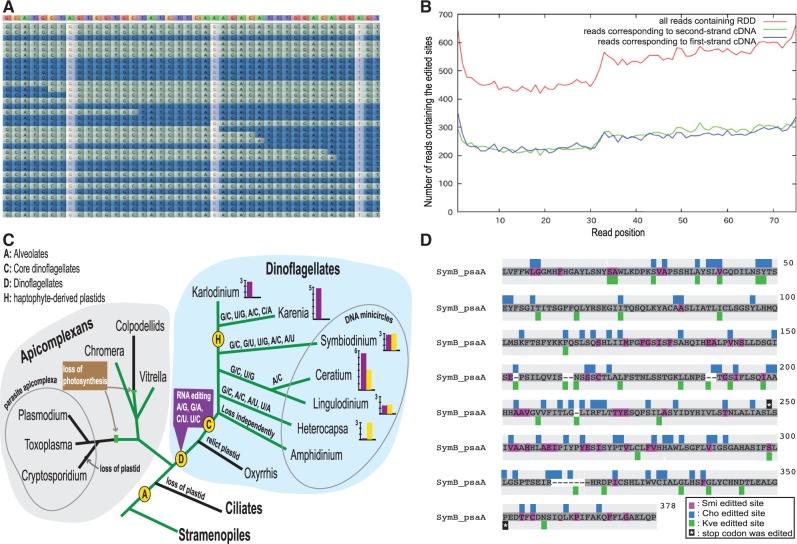
RNA editing in dinoflagellates. (*A*) Screenshot taken from the genome viewer, illustrating both strands of RNA-seq reads mapped on *psbB* genes. Reference DNA bases are colored in the upper part; editing sites are in red; forward reads are in gray; reversed reads are in blue. (*B*) Distribution of RNA editing sites across read positions of plastid genes show no strand or position bias. (*C*) A hypothetical phylogeny showing evolution of alveolate plastid genomes. Green lines indicate photosynthetic organisms. Losses of photosynthetic genes or plastids are indicated. We propose that RNA editing in dinoflagellates occurred after divergence from the alveolate common ancestor or among core dinoflagellates. Ancestral editing types (A/G, G/A, C/U, and U/C) emerged first and then diverse types were generated later in each species. RNA editing was not found in mitochondria of putative basal dinoflagellates, *Oxyrrhis marina* and *Heterocapsa*, but it was found in *Amphidinium*, suggesting that RNA editing may have been lost secondarily in some lineages. Histograms show editing frequency (0.28–6.53%) of plastid mRNA (purple) and rRNA (yellow) at comparable scales among dinoflagellates (Wisecaver and Hackett 2011; Obornik et al. 2012). (*D*) Schematic distribution of RNA editing on aligned, conceptually translated *psaA* genes of dinoflagellates. Amino acid substitutions caused by RNA editing in *Symbiodinium minutum* (Smi), *C. horridum* (Cho), and *K. veneficum* (Kve) are highlighted in pink, blue, and green, respectively. In a comparable region of *psaA*, 78, 52, 30, and 1 edited sites were found in *Ceratium*, *Symbiodinium*, *Karlodinium*, and *Heterocapsa*, respectively. Eighteen sites were shared between *Ceratium* and *Symbiodinium*, eight sites between *Ceratium* and *Karlodinium*, and five sites between *Symbiodinium* and *Karlodinium*. One site was shared between *Heterocapsa* and *Symbiodinium*. However, it was not shown in this figure because it was a 3rd codon-position substitution, which caused no amino acid change in *Heterocapsa*. Asterisks highlighted in black represent stop codons that were corrected by RNA editing. Numbers and locations of RNA editing sites vary greatly among dinoflagellates.

High-coverage reads for both transcriptome and genome, as well as PCR-amplification analyses, enabled us to eliminate sequencing and alignment errors generally produced by high-throughput sequencing (Pickrell et al. 2012). In contrast to RDDs in the human transcriptome (Pickrell et al. 2012), our RNA-seq read alignments of edited sites (fig. 3*A*) were checked manually to confirm that false-positive RDDs from strand, and position biases were not called (fig. 3*B*). Reads mapped incorrectly to other paralogs are unlikely because whole-genome searches showed no duplication of plastid-encoded genes.

In addition, to clarify the possibility of artifacts that could have arisen during generation of the cDNA library, transcriptome assembly generated from independent cDNA libraries made for Roche 454 Sequencer (Bayer et al. 2012) were compared with our results to see whether RNA editing sites from both platforms were consistent. We used ORFs of transcriptome contigs from Illumina assembly (table 1) as queries for BLASTn against the 454 read assembly. The best hit showed more than 99% identity and the transcriptome ID shown in supplementary table S1, Supplementary Material online. Mismatches were mainly from polynucleotide artifacts from the 454 sequencer and uncertain alignments were examined manually to confirm that they were not editing sites. Editing sites found in 454 transcriptome contigs were consistent with our results.

#### RNA Editing Types and Frequency

Accurate mapping of 26,300 Mb of RNA-seq reads to plastid genes revealed at least 471 RNA edits to minicircle genes, but none to nuclear genes. The average frequency of edits per gene was 2.8%, varying from 0.4% in *psbA* to 6.9% in *petD* (table 1). Nine types of RNA editing were found: A/G, C/U, U/C, G/C, A/C, G/A, U/G, G/U, and A/U. The most frequent editing type was A/G (55%), whereas G/A edits comprised only 6.7%. The frequencies of C/U and U/C edits were 13% each. Transversions accounted for only 11.2% of all edits, and G/U, U/G, and A/U transversions were very rare. G/U was a novel type. Interestingly, *petB* and *petD* showed much higher rates of G/C transversion (39% and 24%, respectively).

In total, 364 of 413 of mRNA editing sites (88%) resulted in amino acid substitutions (supplementary table S2, Supplementary Material online). The distribution of substitutions in each gene is shown in supplementary fig. S6, Supplementary Material online). The most frequent substitution was AUN (Ile) to GUN (Val) (62 sites), and the next was ACN (Thr) to GCN (Ala) (40 sites). The frequency of A/G substitutions in the 1st codon position confirms findings of previous dinoflagellate studies (Zauner et al. 2004; Wang and Morse 2006; Dang and Green 2009). Frequencies of substitutions were 1st position (61%), 2nd position (32%), and 3rd position (7%). This contrasts with adenosine to inosine conversion (Bass 2002), which occurs mainly in noncoding human sequences and most targets are found in nervous system transcripts of animals (Rosenthal and Seeburg 2012). We also observed double substitutions at positions 1 and 2 with a frequency of 4.4%, for example, changing from AUG (Met) to GCN (Ala). In *petB* and *petD*, RNA editing even removed premature stop codons, converting them into functional amino acids (supplementary fig. S6, Supplementary Material online), which has been observed in other dinoflagellates (Dorrell and Howe 2012a; Jackson et al. 2013). Information about RNA editing of dinoflagellate plastid genes is still limited. Studies of *Heterocapsa triquetra* (Dang and Green 2009), *Ceratium horridum* (Zauner et al. 2004), *Lingulodinium polyedra* (Wang and Morse 2006), and *Karlodinium veneficum* (Jackson et al. 2013) show different editing types and frequencies (fig. 3*C* and supplementary table S3, Supplementary Material online). *Karlodinium* has haptophyte-derived plastids; in that species, only ancestral editing types (A/G, G/A, C/U, and U/C) have been observed, whereas diverse types (e.g., G/C, A/C, and U/G) seen in other dinoflagellates were absent. However, in haptophyte-derived plastids of *Karenia mikinotoi*, more diverse editing types, consistent with those found in other dinoflagellates, have been found (Dorrell and Howe 2012a). This suggests an early emergence of editing in a common ancestor of dinoflagellates, with diverse editing types arising later (fig. 3*C*). Alignment of conceptually translated sequences with their homologs before and after editing revealed few shared edited sites among them (fig. 3*D* and supplementary table S3, Supplementary Material online). The majority of edited sites appear species specific and occur throughout plastid gene sequences, especially in small genes such as *petD* and *psbE*.

#### What Are the Consequences of RNA Editing?

First, we checked changes in GC content before and after RNA editing, because a majority of RNA editing was from A to G. Of 15,494 total nucleotides within plastid ORFs, including conserved regions of 16 S and 23 S rRNA, %GC content increased from 36.94% to only 38.4% as a result of RNA editing. Therefore, it seems unlikely that the primary function of RNA editing is to increase plastid GC content.

Second, it has been suggested that editing of dinoflagellate plastid gene mRNAs promotes increased identity with homologs of other dinoflagellates (Zauner et al. 2004; Jackson et al. 2013). To further investigate this tendency, identities of conceptual translations of gDNA and cDNA of *Symbiodinium* plastid genes to their homologs in *H. triquetra* were calculated. In *Symbiodinium*, percentage amino acid identity of postedited mRNAs increased by 0–5% depending on the editing frequency (table 1). For instance, percentage identity in *psbA, psbD*, and *psbI* did not change, because these genes possess only 3–8 editing sites. Diverged genes (e.g., *petD*) have higher editing frequencies than more conserved genes (e.g., *psbA*). Interestingly, the amino acid identity of edited *petB* mRNA to its homolog increased by 5%, the highest among the plastid genes, although its editing frequency was only 3.5%. In contrast, the highest editing frequency (6.9%) occurred in *petD*, where the protein identity increased only 3%. This implies that certain editing sites influence protein identity, whereas the remainder are species specific.

**Fig. 4.—:**
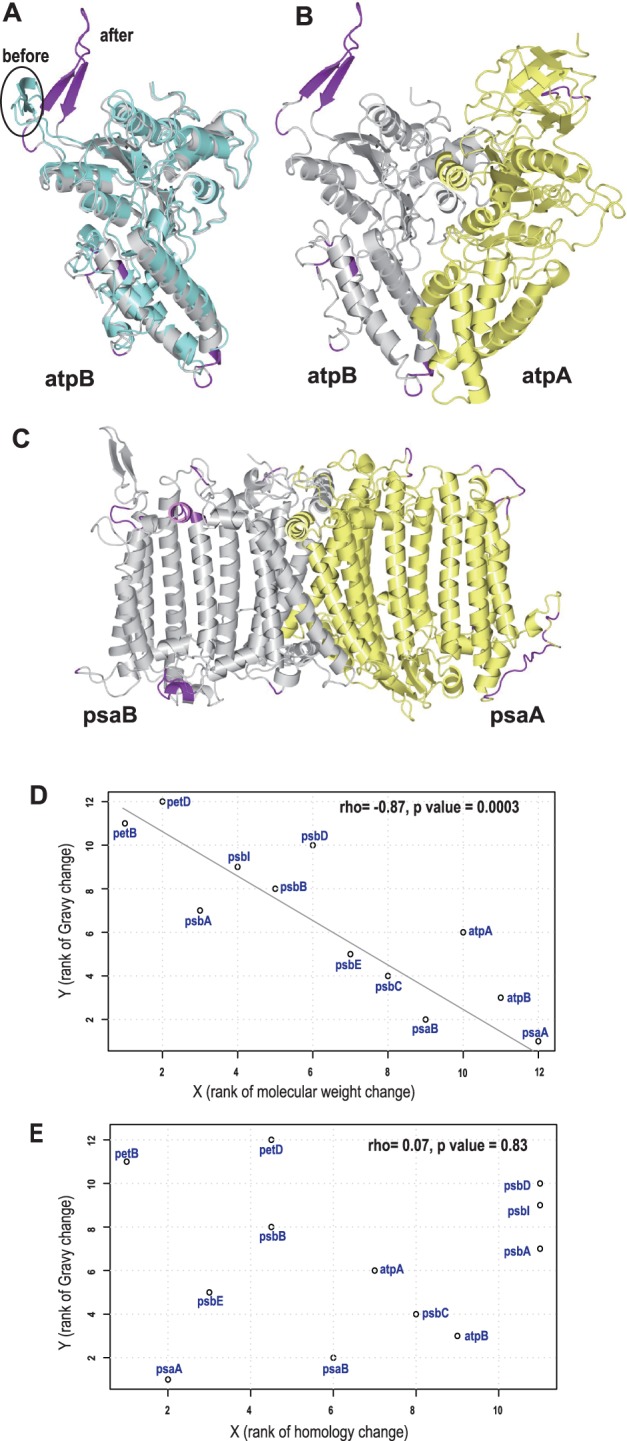
Consequences of RNA editing in *Symbiodinium*. (*A*) Superimposition shows the conformational change in predicted 3D structure of atpB, before (blue) and after (gray) RNA editing. Modeling the interaction of atpA & atpB (*B*) and psaA & psaB (*C*) after RNA editing. Regions in purple show significant conformational changes, leading to functional recovery after editing. (*D*) Rank scatter plot showing correlation between MW and GRAVY of plastid genes after editing (rho = −0.84, *P* value = 0.0003). (*E*) Rank test between protein identity and GRAVY change after editing shows no correlation (rho = 0.07, *P* value = 0.83). RNA editing conserves 3D structure and hydrophobicity in photosystem proteins.

Third, we made 3D structural models of protein products of 11 plastid genes (except psbI, where 3D structure is not available in the SWISS-MODEL database) that showed RNA editing. Of the 361 RNA editing sites, 328 were present in protein secondary structures (supplementary fig. S6 and table S4, Supplementary Material online). Helical structures accounted for 58% (191) of editing sites, whereas 37% and 5% were located on loops or turns, and β-sheet, respectively (supplementary fig. S6 and table S4, Supplementary Material online). Photosystem apparatus proteins are multisubunit membrane protein complexes, in which transmembrane helices are core structures. Stable subunit interactions are essential for these proteins to function. For this reason, we also explored the possibility of conformational changes after RNA editing by in silico analysis, where predicted 3D protein structures before and after editing were compared. At least two proteins, atpB and psaB, (fig. 4*A* and *C*) show high likelihood of functional 3D structural recovery, indicating that RNA editing has an effect on protein conformation. Correct prediction in loop regions is technically difficult; however, RNA editing in these regions may be crucial if it affects protein interactions or alters flexibility of subunit assemblies. When superimposing atpA and atpB 3D (fig. 4*B*) and psaA and psaB 3D (fig. 4*C*) in silico, certain regions (in purple) show significant changes. Therefore, it is likely that preferential RNA editing sites facilitate proper membrane protein structure.

We found that for most plastid-encoded genes, MW diminished after RNA editing. In contrast, average hydropathy, presented by GRAVY score, increased in many of them (supplementary table S5, Supplementary Material online). We confirmed this relationship by performing a Spearman’s rank correlation test between MW and GRAVY after editing (fig. 4*D*), indicating that after RNA editing, molecules tend to become smaller and more hydrophobic (rho = –0.87, *P* value = 0.0003). The next question was whether the hydropathy increase was related to protein identity after editing. A rank test between protein identity and GRAVY change after editing (fig. 4*E*) showed no correlation (rho = 0.07, *P* value = 0.83). Thus, it appears that editing specifically serves to change protein hydropathy.

## Discussion

Phylogenetic analysis of plastid genes of dinoflagellates and other red alga-derived species suggests that a single red algal symbiosis occurred in the common ancestor of the Chromalveolata (Janouskovec et al. 2010). After this acquisition, gene transfer to the host nucleus apparently occurred at some point. Red algal, nuclear-, and plastid-encoded genes in the nuclear genome of the dinoflagellate, *Alexandrium* provide an example (Hackett et al. 2004).

Our analysis revealed a large number of plastid-transfer genes. This apparent relocation of genes raises a number of interesting questions. What selective advantage does this relocation of genes confer? The simplest explanation is that there is a selective advantage to having a compact plastid genome, at least for DNA replication. When transferred to the host, unnecessary genes or genes with redundant functions may have been deleted (Selosse et al. 2001). In principle, endosymbiotic gene transfer may have been accompanied by evolution of host (nuclear) regulatory systems that control symbiont functions. At the same time, the host must coordinate expression of genes distributed between chloroplasts and the nucleus by reading signals that chloroplasts send to control nuclear genes. Processes required for chloroplast function in plants (gene expression, stress signaling, and chloroplast division) are dependent on expression of genes located in the host nucleus (Miyagishima 2011). Many of these genes are originally from symbionts and are likely to be necessary for the establishment of permanent chloroplasts. Therefore, extant genes in different plastid genomes may have been selected via the above processes to arrive at stable genomes in specific environments. At least, comparison of plastid-associated genes of *Symbiodinium* with plastid genomes of related species shows that gene transfer has occurred several times. The loss or transfer of the dinoflagellate chloroplast gene expression machinery occurred following the divergence from apicomplexans and might be associated with the fragmentation and reconfiguration of the dinoflagellate plastid genome to DNA minicircles.

What kinds of genes should be transferred to the nucleus? The most discussed hypothesis, termed colocation for redox regulation (CoRR), claims that the major benefit to retaining core photosystem proteins in plastids is for quick responses to changes in redox potential. Maintenance of redox balance prevents formation of toxic oxygen-free radicals that can react with DNA to cause mutations and that can damage cells (Allen et al. 2011). Recently, a theoretical model proposed that nuclear-encoded peripheral photosystem subunits such as *psbO, psbP, psbU,* and *psaI*, not essential for chloroplast function, allow continuing production of functional proteins, which assist in minimizing oxidative stress resulting from photosynthesis. They may also repair damaged photosystems and reduce levels of reactive oxygen species produced during photosynthesis, thereby having a significant impact on plastid stability (Dorrell and Howe 2012b).

Clearly, after divergence from the alveolate common ancestor, only core photosystem proteins and two rRNA genes remained in the plastid genome of peridinin dinoflagellates. Convergence in organelle genome evolution is shown by the same set of genes for ribosomal proteins having been independently retained by both plastid and mitochondrial genomes (Maier et al. 2013). In dinoflagellate plastid genomes, including that of *Symbiodinium*, all ribosomal proteins have been transferred to the nucleus, and only 16 S and 23 S rRNA have remained in the plastid. This suggests that they are important for ribosome assembly. In *S. minutum*, all core ribosomal proteins (rpl2, rpl14, rpl16, rpl20, and rpl36) (Maier et al. 2013) encompassing the 50 S ribosomal subunit have been transferred to the nucleus. However, rps3, rps7, rps8, rps11, and rps14 that encode core ribosomal proteins, and that bind to 16 S rRNA and form 30 S ribosomal subunits, were not found in the nuclear genome. The loss of these genes may be consistent with reducing the size of 16 S rRNA below the threshold of approximately 1,300 nt reduced to 794 nt (Maier et al. 2013). Deletion mutations of photosystem subunits in cyanobacteria have shown that organisms are unable to grow photoautotrophically if the *psaA, psaB, psaD, psbA-psbF*, and *psbL* genes are deleted, whereas impaired photosynthetic growth or minor phenotypic defects were shown in other *psa* and *psb* mutants (Jansson et al. 1987; Smart et al. 1991; Chitnis 2001). Similar results were shown for *petA, petB*, and *petD* (Kuras and Wollman 1994). Together with the CoRR hypothesis, this suggests that the functional importance of preserving the 12 core photosystem genes in DNA minicircles is to maintain redox balance in *Symbiodinium* chloroplasts. Interestingly, although the plastid genome was being reduced, many transferred plastid-associated genes were duplicated in the nuclear genome. Therefore, the earlier transfer of peripheral photosystem subunits to the nuclear genome and expansion of some sets of genes may have facilitated the establishment of a minimal plastid genome in *Symbiodinium* as oxidative stress was reduced by incorporation into the host.

How then could these 12 core photosystem products be rapidly synthesized in the plastid? Clearly, innovations were required for both nuclear and plastid genes. The complex protein translocation machinery that imports gene products from the cytoplasm into the chloroplast quickly must have been established in dinoflagellates. After divergence from the alveolate ancestor, reduction of the plastid genome to minicircle structures that contain gene coding and control regions, containing at least an origin of replication (Leung and Wong 2009), may have become a common innovation in dinoflagellates to facilitate replication during cell division. Moreover, evolution has driven the cores of dinoflagellate minicircles to diverge greatly among species but to remain extremely conserved within each species (Zhang et al. 2002). This makes it difficult to know what is functionally important.

Because of the lack of a recognizable promoter in minicircle cores, a noncanonical promoter type has been proposed (Zhang et al. 2002; Howe et al. 2008). *Symbiodinium minutum* possesses plastid-transferred genes encoding core subunits of cyanobacterial-type RNA polymerase (fig. 1*A*), which requires sigma factor (*rpoD* gene) for promoter recognition. However, so far, no sigma factor homolog has been found in the *S. minutum* genome nor has a typical eubacterial sigma-type promoter, containing the -35 (TTGaca) and -10 (TAtaaT) consensus sequences. However, a prokaryote-type promoter was found just before putative TSSs of protein-coding genes (fig. 2). This implies that a divergent, sigma-independent promoter was generated during DNA minicircle reconfiguration to compensate for the loss of sigma protein.

A few putative TSSs were also found just before C5 in the cores of *S. minutum* minicircles; therefore, it is possible that more than one type of promoter exists and that different lengths of transcripts need to be processed. In plants, chloroplast polycistronic transcripts possess a specific RNA-binding site for pentatricopeptide repeat (PPR) proteins (Nakamura et al. 2012), right after the TSS, to determine 5′-end maturation by blocking 5′- to 3′-exonucleolytic activity from overtrimming and enhancing protein translation (Luro et al. 2013). In dinoflagellates, poly(U) tract acts to define and protect 3′-end transcripts from degradation and postulated UTR stabilizing factors protect against further cleavage from 5′-exonuclease activity (Barbrook et al. 2012). Therefore, it is likely that the conserved motif (CACCAATGCACC) found right after the putative TSS in DNA minicircles of *S. minutum* might have a similar function. Besides, this motif also contains a eukaryote promoter (CCAAT-box); thus, it may act as a second promoter for ensuring transcription by nuclear RNA polymerase. Through massive gene transfer to the host nucleus, gene regulation is predominately conferred by nuclear genes.

If the cost of redox imbalance were high, innovations would have been required for both the nuclear and plastid genomes to deal with potentially mutated DNA. RNA editing that occurs only in *Symbiodinium* plastid-encoded genes might exist to repair mutations. If that is its function, then where does the information necessary to make corrections reside? It is hard to believe that the emergence of RNA editing is just for DNA correction, because editing occurs mainly in the first codon position, which causes amino acid substitutions. Editing biased toward a single codon position is unlikely to occur at random. So far, dinoflagellate data, including the present results, show that dinoflagellates share a common gene repertoire but that editing sites and frequencies are species-specific. Perhaps these species-specific differences are associated with varied environmental conditions (Georg et al. 2010) and it may be that photosynthetic activity varies between symbiotic and free-living dinoflagellates. It appears that RNA editing in plastids serves primarily to restore evolutionarily conserved amino acids and hydrophobicity of core photosystem molecules. This promotes proper folding and stable interactions of protein subunits (Yura and Go 2008). It has been shown that RNA editing in land plants is crucial for assembly of the whole-cytochrome b6f complex. Introducing a modified petB of *C. reinhardtii*, having proline at residue 204 instead of leucine, mimics the unedited codon found in land plants. This mutant showed defective electron transfer due to a decrease in cytochrome b6f subunits (Zito et al. 1997).

*Symbiodinium* RNA editing mainly occurs in helices that comprise the structural core of transmembrane proteins. In general, mutations in this region could have a significant impact on protein stability. Most conformational changes after RNA editing of atpB protein appeared in the β-sheet, which may be involved in interaction with other atp protein subunits. Structural analysis of the photosystem I reaction center between plants and cyanobacteria showed that the backbone conformation and side chains of the core complex are highly similar. However, comparison at the atomic level showed major functional and structural differences between them (Amunts and Nelson 2008). Therefore, slight conformational changes of psaB after RNA editing of *Symbiodinium* may have significant effects. To verify this, more comprehensive structural analysis is yet required. Overall, our results suggested that plastid gene RNA editing may simultaneously increase plasticity of core photosystem proteins (green proteins, fig. 1*b*), while preserving quaternary structure necessary for initiation of photosystem complex assembly.

The absence of RNA editing in the plastid genome of *C. velia*, belonging to a sister group of dinoflagellates, was reported recently (Janouskovec et al. 2013). This suggests that the innovation of RNA editing in dinoflagellates likely occurred after divergence from the alveolate common ancestor (fig. 3). On the other hand, RNA editing was not found in the plastid genome of *Amphidinium operculatum* (Barbrook et al. 2001) or in mitochondria of the putative basal dinoflagellate, *Oxyrrhis marina* (Zhang et al. 2008). Therefore, if the value of RNA editing is high, then what do these other organisms use to deal with similar situations? Why are so many types of editing required in dinoflagellates? What mechanism is responsible for this editing? So far very different RNA editing mechanisms have been reported in mammals and plants. Although animals use enzymatic base conversion, plant organelles use an RNA-binding protein, PPR (Schmitz-Linneweber and Small 2008). In *Trypanosoma brucei*, 28 unique PPR proteins have been found, and they play essential roles in mitochondrial rRNA biogenesis and stability (Pusnik et al. 2007). In *Karenia brevis*, approximately 100 annotated PPR proteins have been found (Morey et al. 2011), and their expression increased 3-fold within 1 hr after addition of nutrients. In the *S. minutum* genome, one ADAR gene and approximately 620 PPR genes were present in http://marinegenomics.oist.jp/genomes/gallery/ (last accessed June 16, 2014) (Koyanagi et al. 2013). Interestingly, two PPR genes in *S. minutum* contain a DYW motif, which has a role in C/T editing in *Arabidopsis* because a DYW motif matches the active site of cytidine deaminases (Salone et al. 2007). Thus, it is possible that multiple systems perform different types of editing, as suggested for mitochondrial mRNA editing in dinoflagellates (Lin et al. 2002), and it is highly likely that PPR proteins are involved in diverse types of RNA editing in *Symbiodinium*. Although the function of PPR proteins is not known in dinoflagellates, their high duplication levels and the presence of spliced leader sequences suggest an important role in posttranscriptional control or RNA processing (Salone et al. 2007).

Convergent evolution has been a factor in the development of many important traits, such as common odd features (structure of organellar genomes, RNA editing and processing, nuclear polycistronic transcription, and spliced leaders) between the euglenozoans and the alveolates (Lukes et al. 2009). It is the process whereby distant lineages independently evolve similar traits as a result of adaption to similar environments (Doolittle 1994). The RNA editing type and pattern in *Symbiodinium* is very different from that of land plants; however, both have had similar consequences. Clearly, C/U editing at the second codon position has occurred mainly in plant organelles to increase the frequency of leucine, which is highly hydrophobic (Yura and Go 2008). In dinoflagellate organelles, including *Symbiodinium*, nine types of editing operate at the first codon position to increase molecular hydropathy. This implies that similar environments in organelles may compel RNA editing to maintain a similar outcome.

## Supplementary Material

Supplementary figures S1–S6 and tables S1–S5 are available at *Genome Biology and Evolution* online (http://www.gbe.oxfordjournals.org/).

Supplementary Data
