# Supplementary material for: Massive Gene Transfer and Extensive RNA Editing of a Symbiotic Dinoflagellate Plastid Genome
Source: Genome Biol Evol. 2014 May 31;6(6):1408–22. doi: 10.1093/gbe/evu109 (PMC4079212; doi:10.1093/gbe/evu109)
Supplement: Supplementary Data [file supp_6_6_1408__index.html]

Massive gene transfer and extensive RNA editing of a symbiotic dinoflagellate plastid genome — Massive Gene Transfer and Extensive RNA Editing of a Symbiotic Dinoflagellate Plastid Genome — Supplementary Data 

# Massive Gene Transfer and Extensive RNA Editing of a Symbiotic Dinoflagellate Plastid Genome

## Supplementary Data

files

**Files in this Data Supplement:**

- Supplementary Data - pdf file
- Supplementary Data - pdf file
