# Supplementary material for: Massive Gene Transfer and Extensive RNA Editing of a Symbiotic Dinoflagellate Plastid Genome
Source: Genome Biol Evol. 2014 May 31;6(6):1408–22. doi: 10.1093/gbe/evu109 (PMC4079212; doi:10.1093/gbe/evu109)
Supplement: Supplementary Data [file supp_evu109_suppFigGBE.pdf]

SUPPLEMENTARY FIGURES

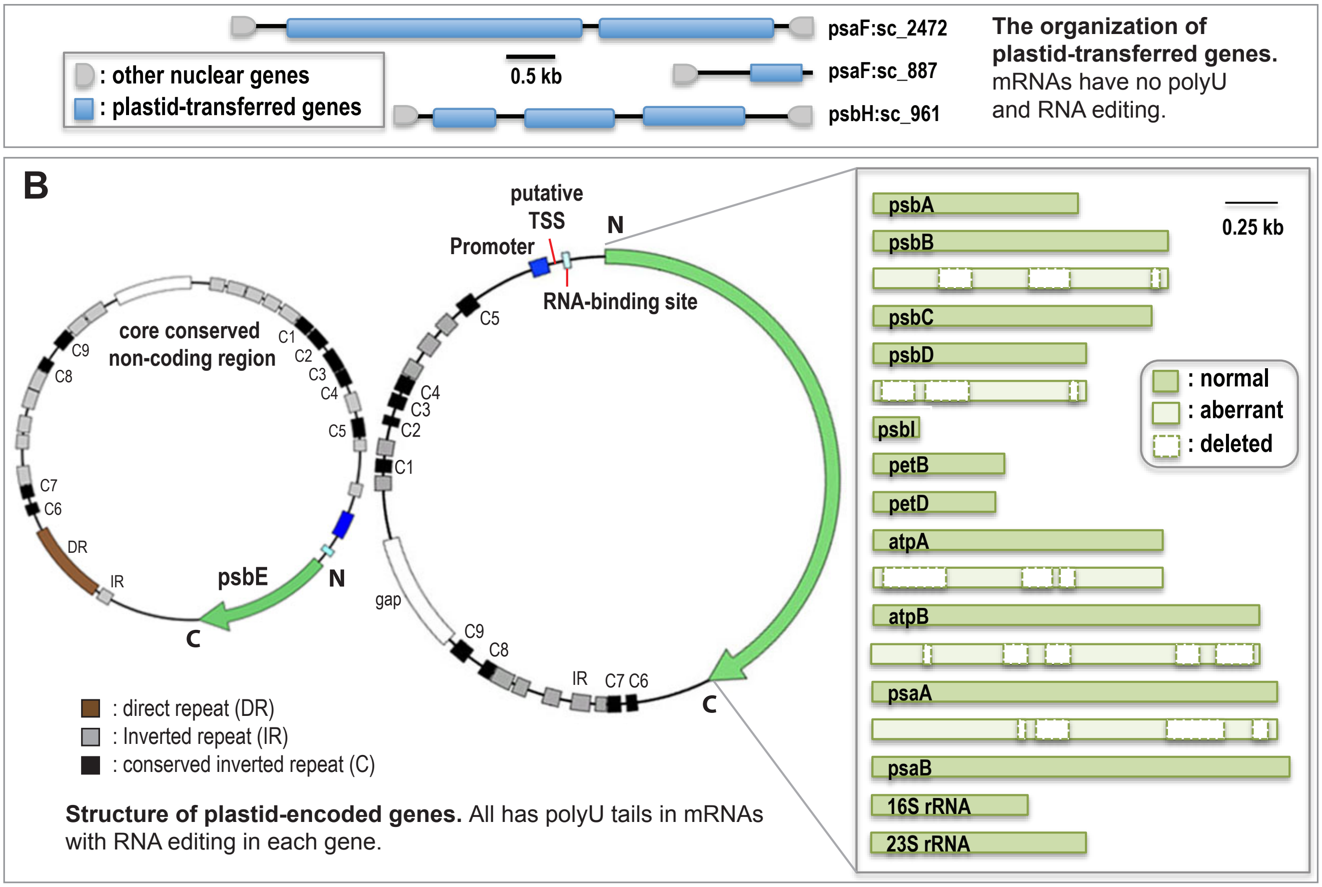

Supplementary Figure S1: Structure of plastid-transferred genes and plastid-encoded genes (DNA minicircles) of *Symbiodinium minutum*. (**A**) Two examples of plastid-transferred genes. Two genes show duplications, often adjacent, on the same scaffold, and only rarely on different scaffolds. (**B**) Fourteen plastid-encoded genes organized in minicircles. The most common minicircle structure contains a coding region, plus a non-coding region containing a high density of inverted repeats (IR) (gray) and a minimum of nine conserved regions (black). The number of IRs varies from 10-16. In coding regions, aberrant forms were found in *psbB, psbD, atpA, atpB* and *psaA* with 3-5 deletions of ~50-300 bp. The *psbE* mincircle contains a direct repeat (brown) of 35 bp (GCAATCCTGCAGCATA**GCA**TATGCTGCAACCTGCT) in 4 copies. Each repeat is inverted. Therefore, *psbE* has the highest density of IRs (29) observed in *Symbiodinium*. A "GAP box" (white) represents incomplete sequences that were estimated from PCR products. A comparison of (A) and (B) above, demonstrates that nuclear-encoded genes have a completely different structure from plastid-encoded genes.

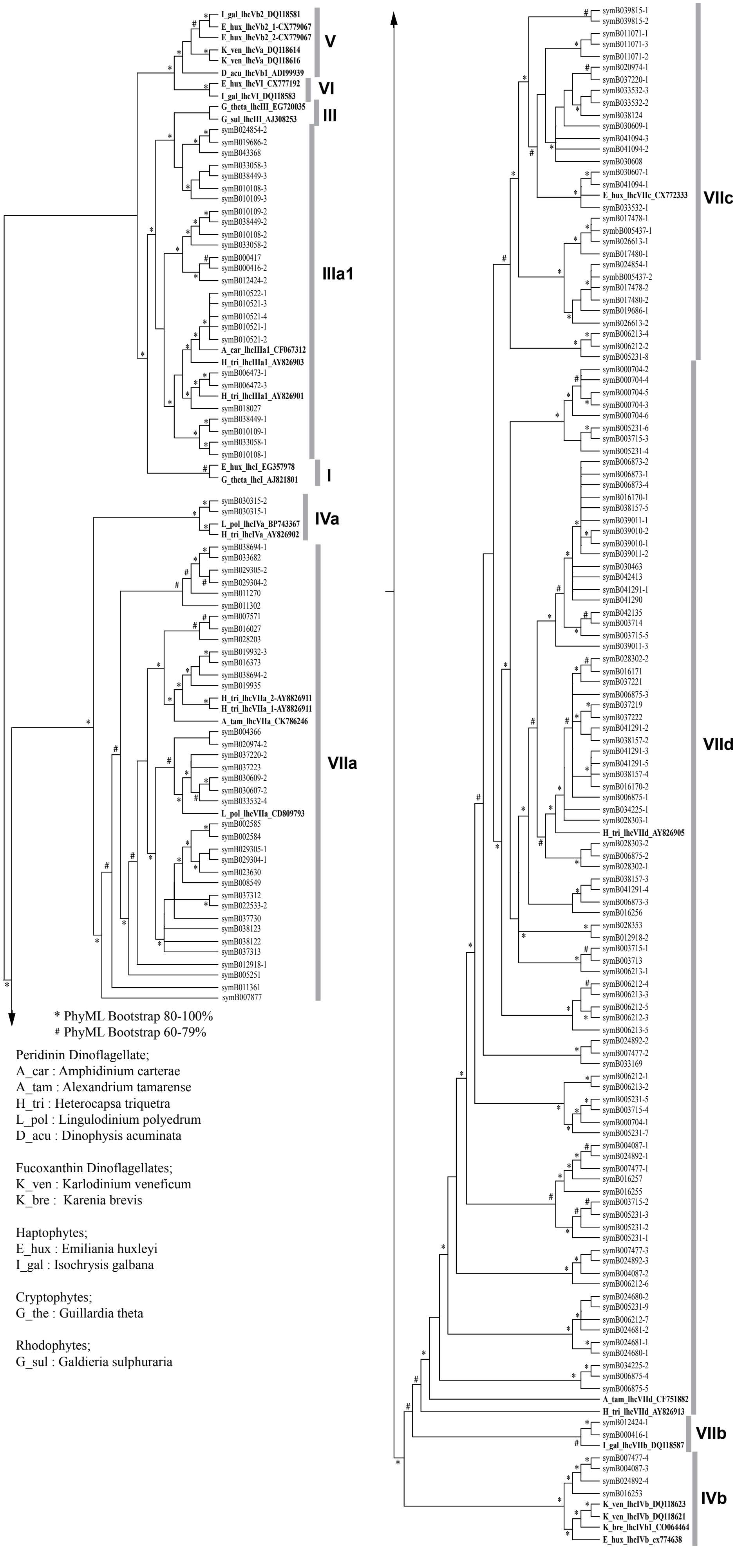
V
VI
III
IIIa1
I
IVa
VIIa
VIIc
VIId
VIIb
IVb
* PhyML Bootstrap 80-100%
# PhyML Bootstrap 60-79%
Peridinin Dinoflagellate;
A_car : Amphidinium carterae
A_tam : Alexandrium tamarense
H_tri : Heterocapsa triquetra
L_pol : Lingulodinium polyedrum
D_acu : Dinophysis acuminata
Fucoxanthin Dinoflagellates;
K_ven : Karlodinium veneficum
K_bre : Karenia brevis
Haptophytes;
E_hux : Emiliania huxleyi
I_gal : Isochrysis galbana
Cryptophytes;
G_the : Guillardia theta
Rhodophytes;
G_sul : Galdieria sulphuraria

Supplementary Figure S2: Phylogenetic tree analysis of light-harvesting complexe proteins (LHCs) of *Symbodinium minutum*. Maximum likelihood analysis based on amino acid sequences of 101 *lhcb* genes. Polyprotein LHC sequences are treated as separate sequences with specific ID numbers. LHC sequences classified by Hoffman *et al*. (bold) were used as references to define *Symbiodinum* LHCs into subfamilies. By their share number, it can be seen that proteins in subfamily VIId have undergone extensive duplication in *Symbiodinium*.

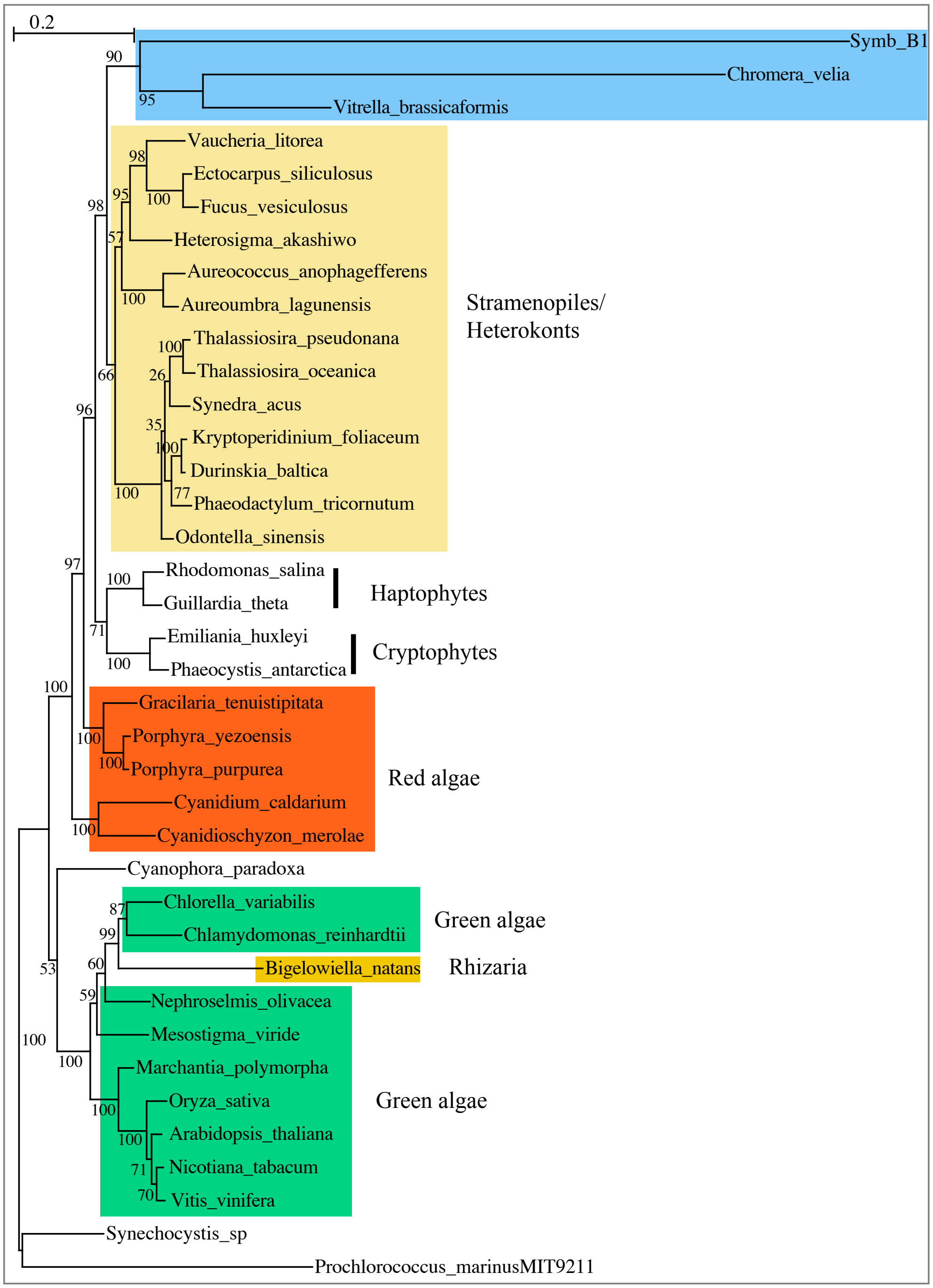

Supplementary Figure S3: Genome-wide molecular phylogenetics of *Symbodinium minutum* based on proteins encoded by plastid DNA minicircles. Maximum-likelihood analysis based on alignment of 3,789 amino acid residues from proteins encoded by 11 core photosystem genes. *psbI* was excluded because it has been lost from the *Chromera* plastid genome. The clade of phylum Chromalveolata/red algae is supported by a 100% bootstrap value, whereas the Group Rhizaria is associated with green algae, forming an independent clade. The scale bar represents 0.2 expected substitutions per site in the aligned regions. The dinoflagellates and apicomplexans (blue) form a clade with the red algae (red) and the stramenopiles (yellow).

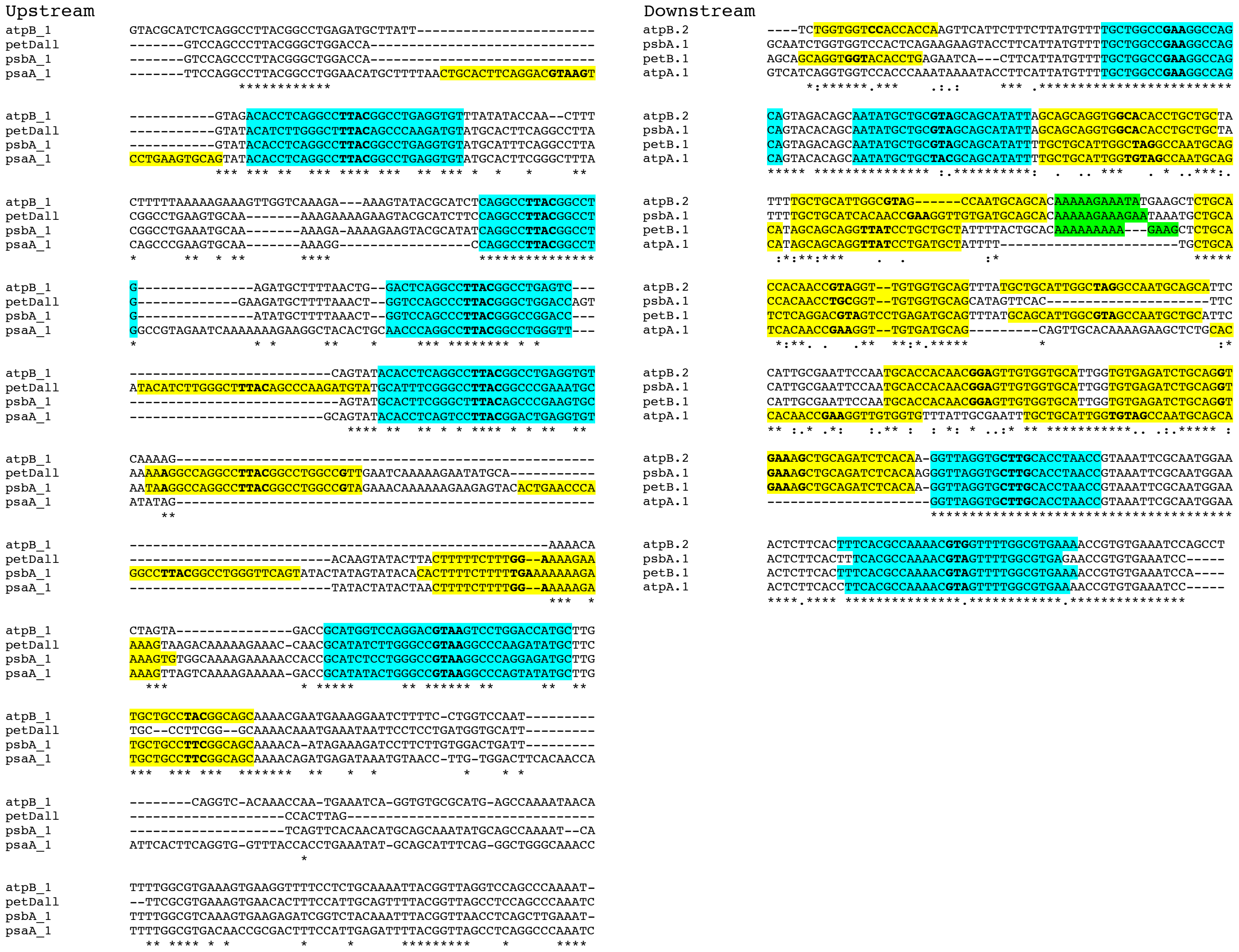

Supplementary Figure S4: Alignment of non-coding sequences of *Symbiodinium minutum* minicircles. Since the sequence of the non-coding region was incomplete, the upstream sequence beyond the start codon and the downstream sequence after the stop codon were used for the alignment. Inverted repeats (IRs) contain conserved regions (blue) and variable regions (yellow). Conserved regions are embedded in variable regions, which contain insertions and deletions. These variable regions also contain many IRs. Bold bases are intra-IR spacers. The number of IRs varies in the variable region. A region of high AT-content (green), is surrounded by an IR region. All *Symbiodinium* minicircles contain large numbers of IRs, the function of which is presently unknown. However, it has been suggested that the region bracketed by these sequences may contain the origin of replication.

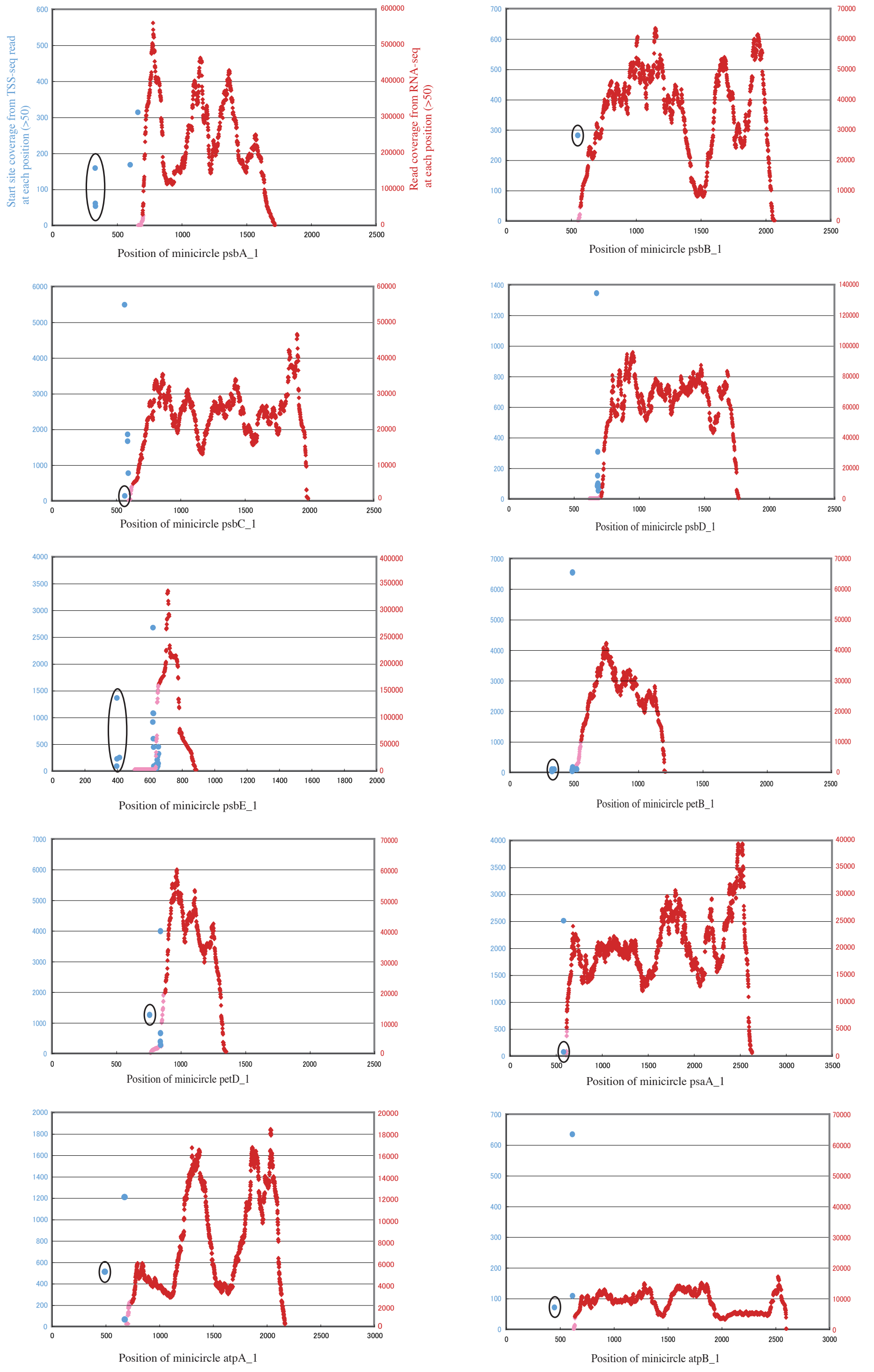

Supplementary Figure S5: TSS analysis of protein-coding genes of *Symbiodinium minutum* DNA minicircles. Blue dots show start site coverage (left Y axis) of reads from the TSS-library. Start site coverage of ORFs is omitted. In contrast, pink dots on UTRs and red dots on ORFs indicate read coverage (right Y axis) from RNA-seq without enzyme treatments. Most 5' dots removed from ORFs are surrounded by black line, corresponding to putative TSS sites (fig. 2). For psbD-containing minicircle, RNA-seq reads without enzyme treatments are mapped on most 5' region for that upstream of ORF.

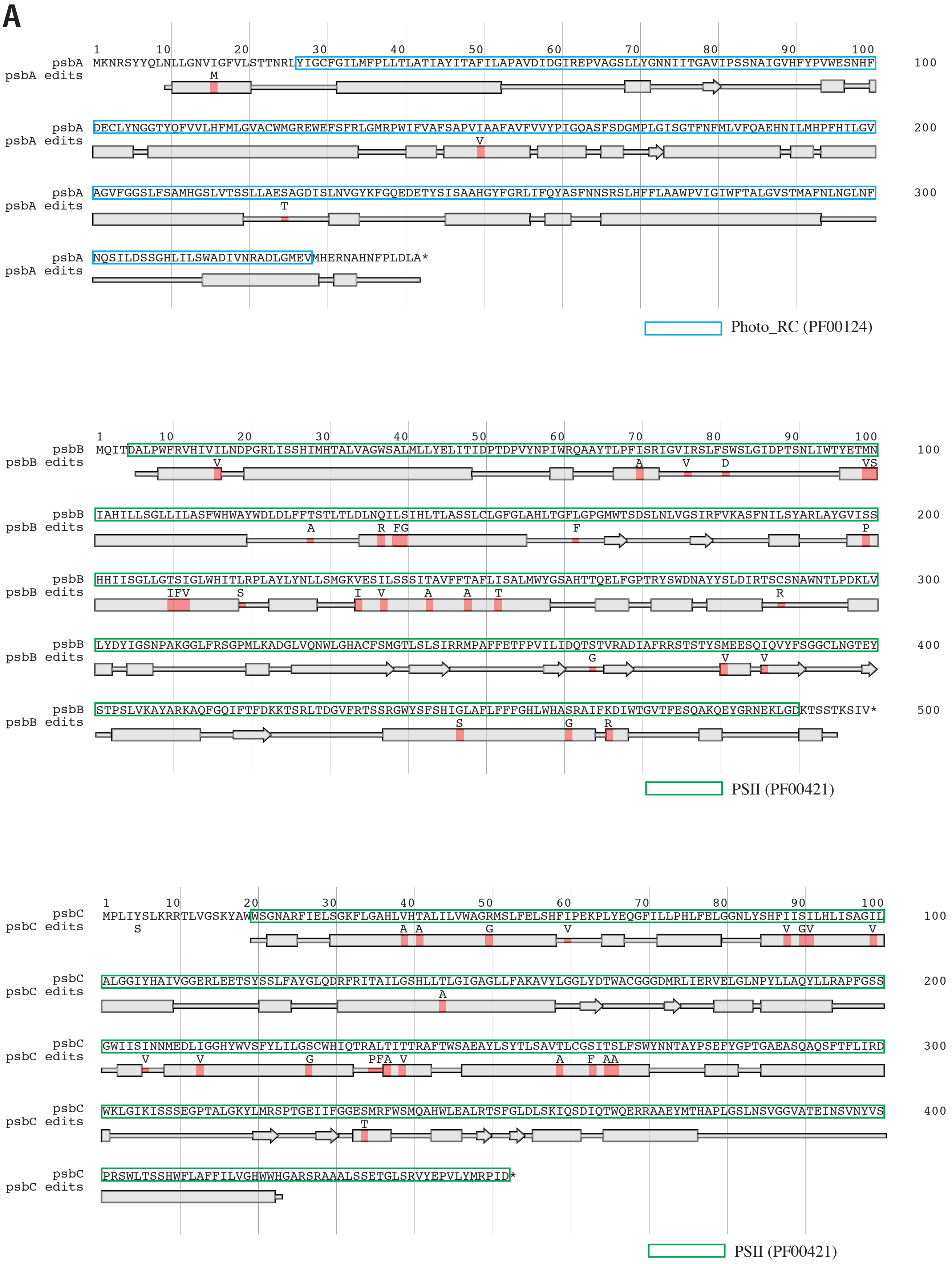
A
psbA MKNRSYYQLNLLGNVIGFVLSTTNRLYIGCFGILMFPLLTLATIAYITAFILAPAVDIDGIREPVAGSLLYGNNIITGAVIPSSNAIGVHFYPVWESNHF 100
psbA edits M
psbA DECLYNGGTYQFVVLHFMLGVACWMGREWEFSFRLGMRPWIFVAFSAPVIAAFAVFVVYPIGQASFSDGMPLGISGTFNFMLVFQAEHNILMHPFHILGV 200
psbA edits V
psbA AGVFGGSLFSAMHGSLVTSSLLAESAGDISLNVGYKFGQEDETYSISAAHGYFGRLIFQYASFNNSRSLHFFLAAWPVIGIWFTALGVSTMAFNLNGLNF 300
psbA edits T
psbA NQSILDSSGHLILSWADIVNRADLGMEVMHERNAHNFPLDLA*
psbA edits
Photo_RC (PF00124)
psbB MQITDALPWFRVHIVILNDPGRLISSHIMHTALVAGWSALMLLYELITIDPTDPVYNPIWRQAAYTLPFISRIGVIRSLFSWSLGIDPTSNLIWTYETMN 100
psbB edits V A V D VS
psbB IAHILLSGLLILASFWHWAYWDLDLFFTSTLTLDLNQILSIHLTLASSLCLGFGLAHLTGFLGPGMWTSDSLNLVGSIRFVKASFNILSYARLAYGVISS 200
psbB edits A R FG F P
psbB HHIISGLLGTSIGLWHITLRPLAYLYNLLSMGKVESILSSSITAVFFTAFLISALMWYGSAHTTQELFGPTRYSWDNAYYSLDIRTSCSNAWNTLPDKLV 300
psbB edits IFV S I V A A T R
psbB LYDYIGSNPAKGGLFRSGPMLKADGLVQNWLGHACFSMGTLSLSIRRMPAFFETFPVILIDQTSTVRADIAFRRSTSTYSMEESQIQVYFSGGCLNGTEY 400
psbB edits G V V
psbB STPSLVKAYARKAQFGQIFTFDKKTSRLTDGVFRTSSRGWYSFSHIGLAFLFFFGHLWHASRAIFKDIWTGVTFESQAKQEYGRNEKLGDKTSSTKSIV* 500
psbB edits S G R
PSII (PF00421)
psbC MPLIYSLKRRTLVGSKYAWWSGNARFIELSGKFLGAHLVHTALILVWAGRMSLFELSHFIPEKPLYEQGFILLPHLFELGGNLYSHFIISILHLISAGIL 100
psbC edits S A A G V V GV V
psbC ALGGIYHAIVGGERLEETSYSSLFAYGLQDRFRITAILGSHLLTLGIGAGLLFAKAVYLGGLYDTWACGGGDMRLIERVELGLNPYLLAQYLLRAPFGSS 200
psbC edits A
psbC GWIISINNMEDLIGGHYWVSFYLILGSCWHIQTRALTITTRAFTWSAEAYLSYTLSAVTLCGSITSLFSWYNNTAYPSEFYGPTGAEASQAQSFTFLIRD 300
psbC edits V V G PFA V A F AA
psbC WKLGIKISSSEGPTALGKYLMRSPTGEIIFGGESMRFWSMQAHWLEALRTSFGLDLSKIQSDIQTWQERRAAEYMTHAPLGSLNSVGGVATEINSVNYVS 400
psbC edits T
psbC PRSWLTSSHWFLAFFILVGHWWHGARSRAAALSSETGLSRVYEPVLYMRPID*
psbC edits
PSII (PF00421)

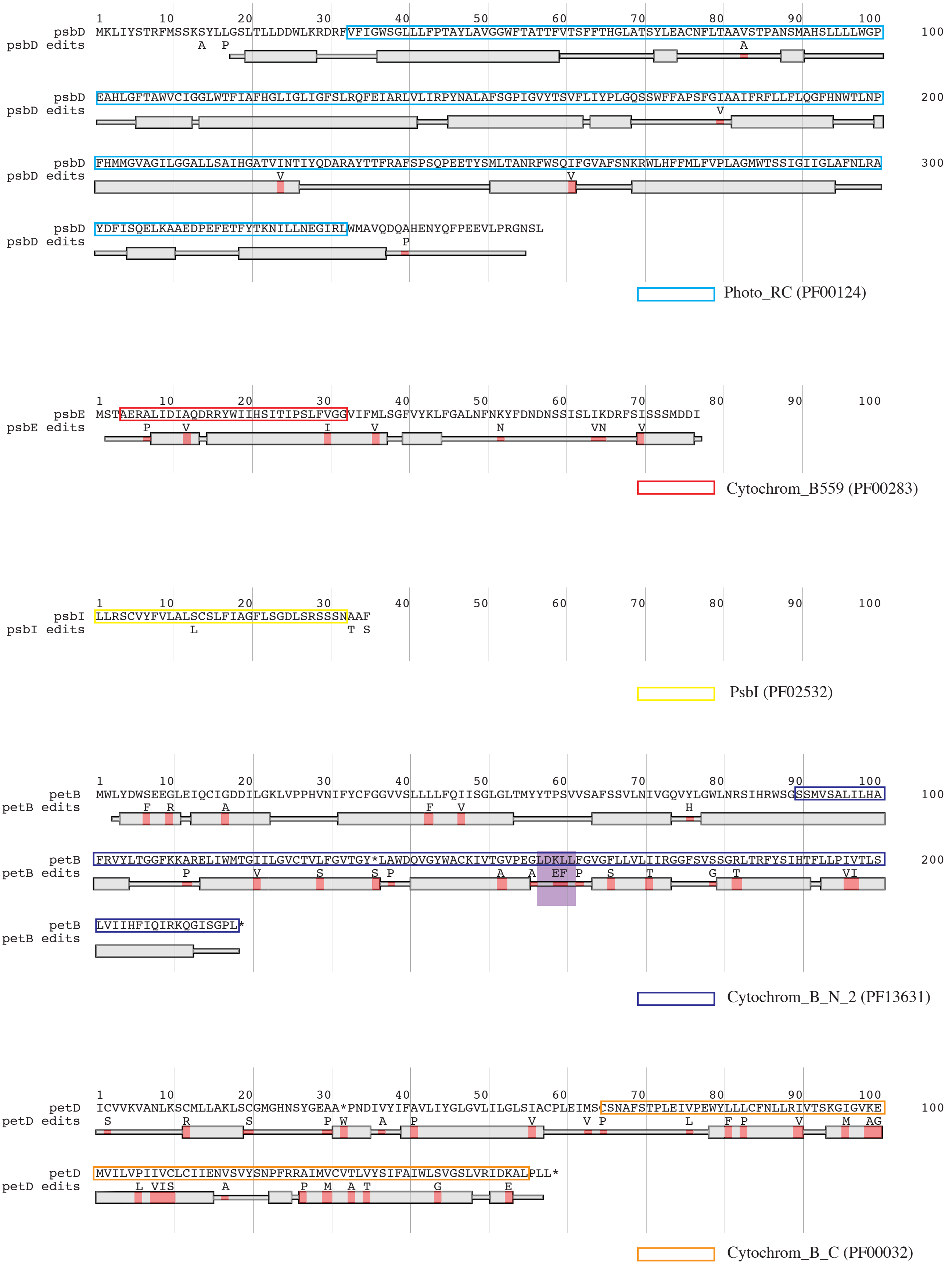

psbD MKLIYSTRFMSSKSYLLGSLTLLDDWLKRDRFVFIGWSGLLLFPTAYLAVGGWFTATTFVTSFFTHGLATSYLEACNFLTAAVSTPANSMAHSLLLLWGP 100
psbD edits A P A
psbD EAHLGFTAWVCIGGLWTFIAFHGLIGLIGFSLRQFEIARLVLIRPYNALAFSGPIGVYTSVFLIYPLGQSSWFFAPSFGIAAIFRFLLFLQGFHNWTLNP 200
psbD edits V
psbD FHMMGVAGILGGALLSAIHGATVINTIYQDARAYTTFRAFSPSQPEETYSMLTANRFWSQIFGVAFSNKRWLHFFMLFVPLAGMWTSSIGIIGLAFNLRA 300
psbD edits V V
psbD YDFISQELKAAEDPEFETFYTKNILLNEGIRLWMAVQDQAHENYQFPEEVLPRGNSL
psbD edits P
Photo_RC (PF00124)
psbE MSTAERALIDIAQDRRYWIIHSITIPSLFVGGVIFMLSGFVYKLFGALNFNKYFDNDNSSISLIKDRFSISSSMDDI
psbE edits P V I V N VN V
Cytochrom_B559 (PF00283)
psbI LLRSCVYFVLALSCSLFIAGFLSGDLSRSSSNAAF
psbI edits L T S
PsbI (PF02532)
petB MWLYDWSEEGLEIQCIGDDILGKLVPPHVNIFYCFGGVVSLLLLFQIISGLGLTMYYTPSVVSAFSSVLNIVGQVYLGWLNRSIHRWSGSSMVSALILHA 100
petB edits F R A F V H
petB FRVYLTGGFKKARELIWMTGIILGVCTVLFGVTGY*LAWDQVGYWACKIVTGVPEGLDKLLFGVGFLLVLIIRGGFSVSSGRLTRFYSIHTFLLPIVTLS 200
petB edits P V S S P A A EF P S T G T VI
petB LVIIHFIQIRKQGISGPL*
Cytochrom_B_N_2 (PF13631)
petD ICVVKVANLKSCMLLAKLSCGMGHNSYGEAA*PNDIVYIFAVLIYGLGVLILGLSIACPLEIMSCSNAFSTPLEIVPEWYLLLCFNLLRIVTSKGIGVKE 100
petD edits S R S P W A P V V P L F P V M AG
petD MVILVPIIVCLCIIENVSVYSNPFRRAIMVCVTLVYSIFAIWLSVGSLVRIDKALPLL*
petD edits L VIS A P M A T G E
Cytochrom_B_C (PF00032)

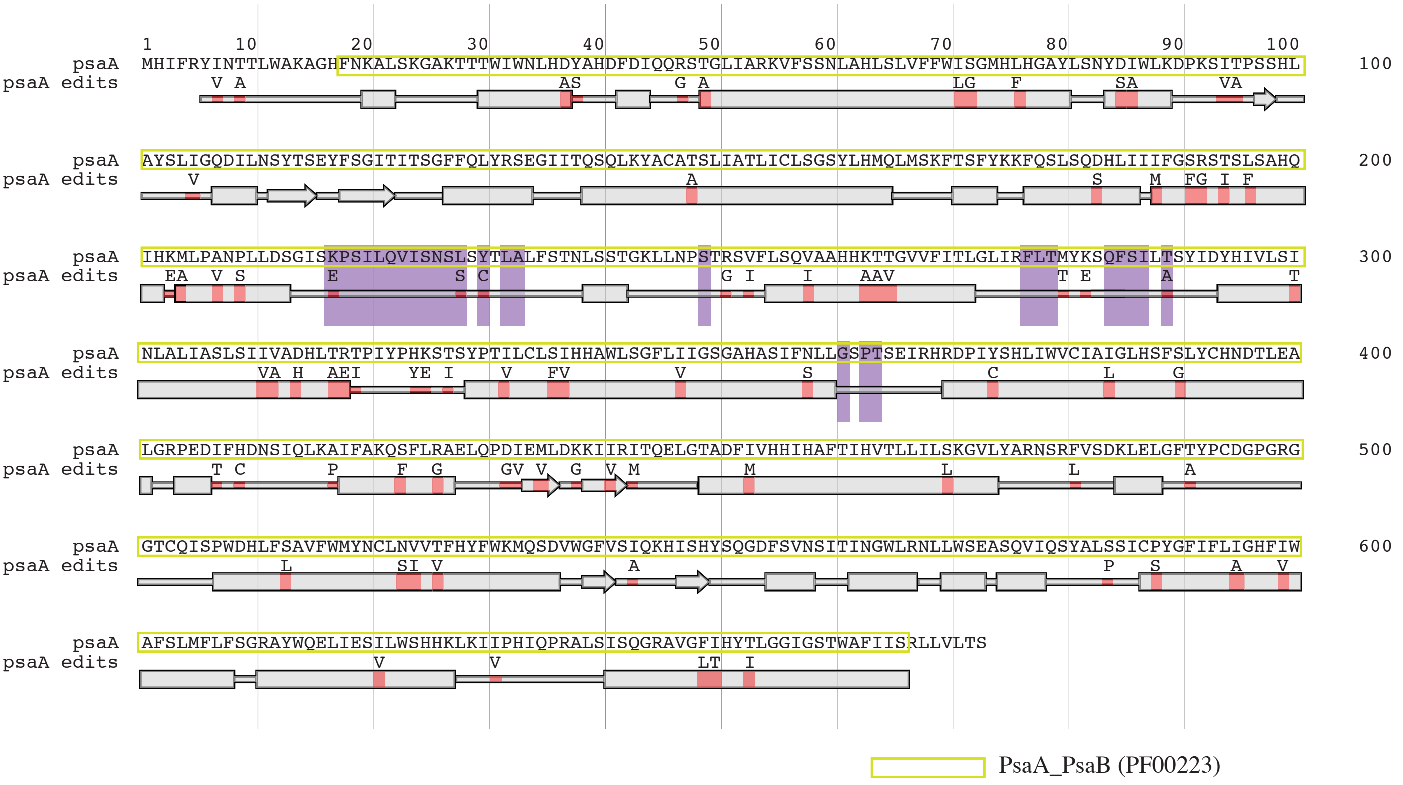

1 10 20 30 40 50 60 70 80 90 100
psaA MHIFRYINTTLWAKAGHFNKALSKGAKTTTWIWNLHDYAHDFDIQQRSTGLIARKVFSSNLAHLSLVFFWISGMHLHGAYLSNYDIWLKDPKSITPSSHL 100
psaA edits V A AS G A LG F SA VA
psaA AYSLIGQDILNSYTSEYFSGITITSGFFQLYRSEGIITQSQLKYACATSLIATLICLSGSYLHMQLMSKFTSFYKKFQSLSQDHLIIIFGSRSTSLSAHQ 200
psaA edits V A S M FG I F
psaA IHKMLPANPLLDSGISKPSILQVISNSLSYTLALFSTNLSSTGKLLNPSTRSVFLSQVAAHHKTTGVVFITLGLIRFLTMYKSQFSILTSYIDYHIVLSI 300
psaA edits EA V S E S C G I I AAV T E A T
psaA NLALIASLSIIVADHLTRTPIYPHKSTSYPTILCLSIHHAWLSGFLIIGSGAHASIFNLLGSPTSEIRHRDPIYSHLIWVCIAIGLHSFSLYCHNDTLEA 400
psaA edits VA H AEI YE I V FV V S C L G
psaA LGRPEDIFHDNSIQLKAIFAKQSFLRAELQPDIEMLDKKIIRITQELGTADFIVHHIHAFTIHVTLLILSKGVLYARNSRFVSDKLELGFTYPCDGPGRG 500
psaA edits T C P F G GV V G V M M L L A
psaA GTCQISPWDHLFSAVFWMYNCLNVVTFHYFWKMQSDVWGFVSIQKHISHYSQGDFSVNSITINGWLRNLLWSEASQVIQSYALSSICPYGFIFLIGHFIW 600
psaA edits L SI V A P S A V
psaA AFSLMFLFSGRAYWQELIESILWSHHKLKIIPHIQPRALSISQGRAVGFIHYTLGGIGSTWAFIISRLLVLTS
psaA edits V V LT I
PsaA_PsaB (PF00223)

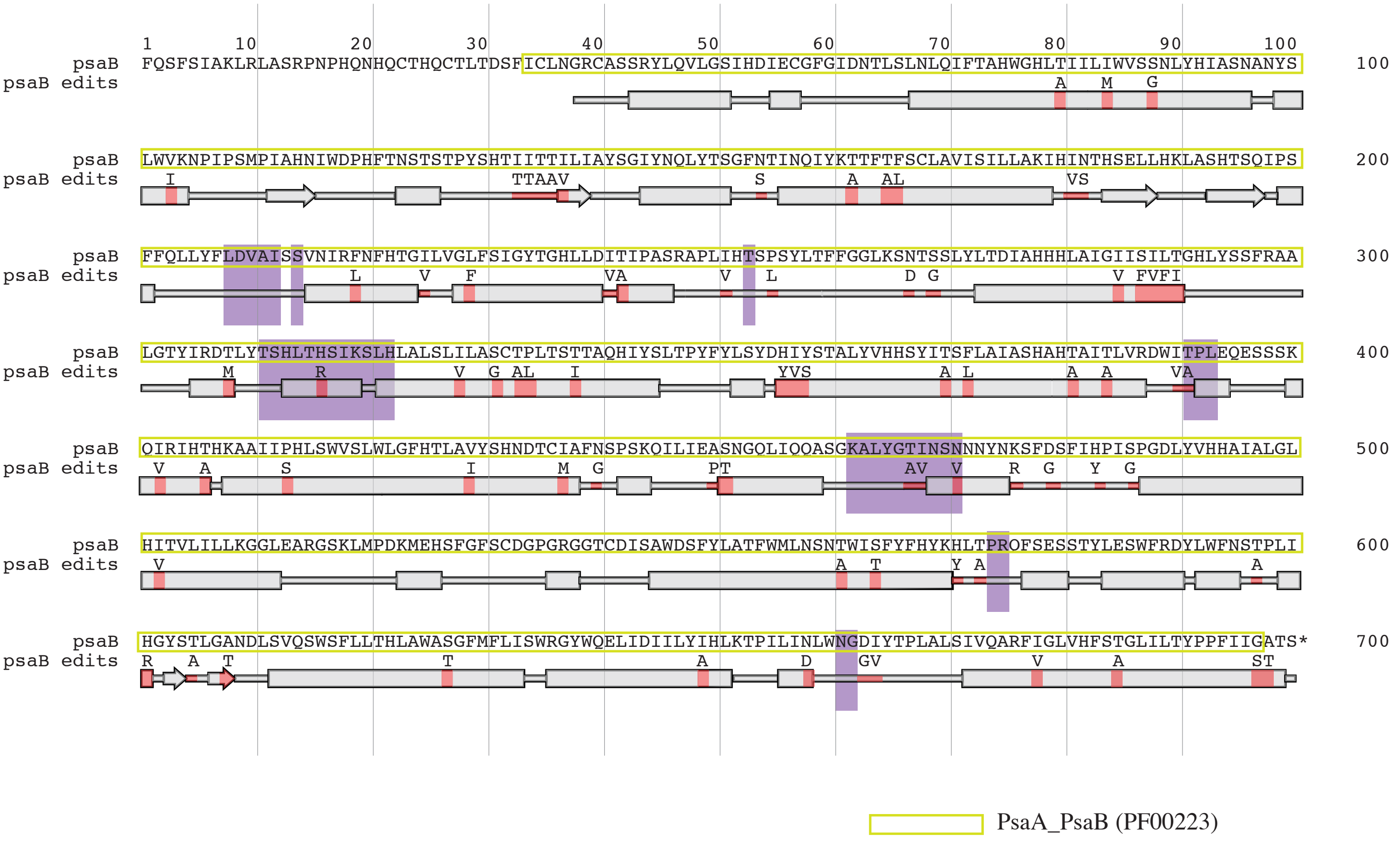

1 10 20 30 40 50 60 70 80 90 100
psaB FQSFSIAKLRLASRPNPHQNHQCTHQCTLTDSFICLNGRCASSRYLQVLGSIHDIECGFGIDNTLSLNLQIFTAHWGHLTIILIWVSSNLYHIASNANYS 100
psaB edits A M G
psaB LWVKNPIPSMPIAHNIWDPHFTNSTSTPYSHTIITTILIAYSGIYNQLYTSGFNTINQIYKTTFTFSCLAVISILLAKIHINTHSELLHKLASHTSQIPS 200
psaB edits I TTAAV S A AL VS
psaB FFQLLYFLDVAISSVNIRFNFHTGILVGLFSIGYTGHLLDITIPASRAPLIHTSPSYLTFFGGLKSNTSSLYLTDIAHHHLAIGIISILTGHLYSSFRAA 300
psaB edits L V F VA V L D G V FVFI
psaB LGTYIRDTLYTSHLTHSIKSLHLALSLILASCTPLTSTTAQHIYSLTPYFYLSYDHIYSTALYVHHSYITSFLAIASHAHTAITLVRDWITPLEQESSSK 400
psaB edits M R V G AL I YVS A L A A VA
psaB QIRIHTHKAAIIPHLSWVSLWLGFHTLAVYSHNDTCIAFNSPSKQILIEASNGQLIQQASGKALYGTINSNNNYNKSFDSFIHPISPGDLYVHHAIALGL 500
psaB edits V A S I M G PT AV V R G Y G
psaB HITVLILLLKGGLEARGSKLMPDKMEHSFGFSCDGPGRGGTCDISAWDSFYLATFWMLNSNTWISFYFHYKHLTPROFSESSTYLESWFRDYLWFNSTPLI 600
psaB edits V A T Y A A
psaB HGYSTLGANDLSVQSWSFLLTHLAWASGFMFLISWRGYWQELIDIILYIHLKTPILINLWNGDIYTPLALSIVQARFIGLVHFSTGLILTYPPFIIGATS* 700
psaB edits R A T T A D GV V A ST
PsaA_PsaB (PF00223)

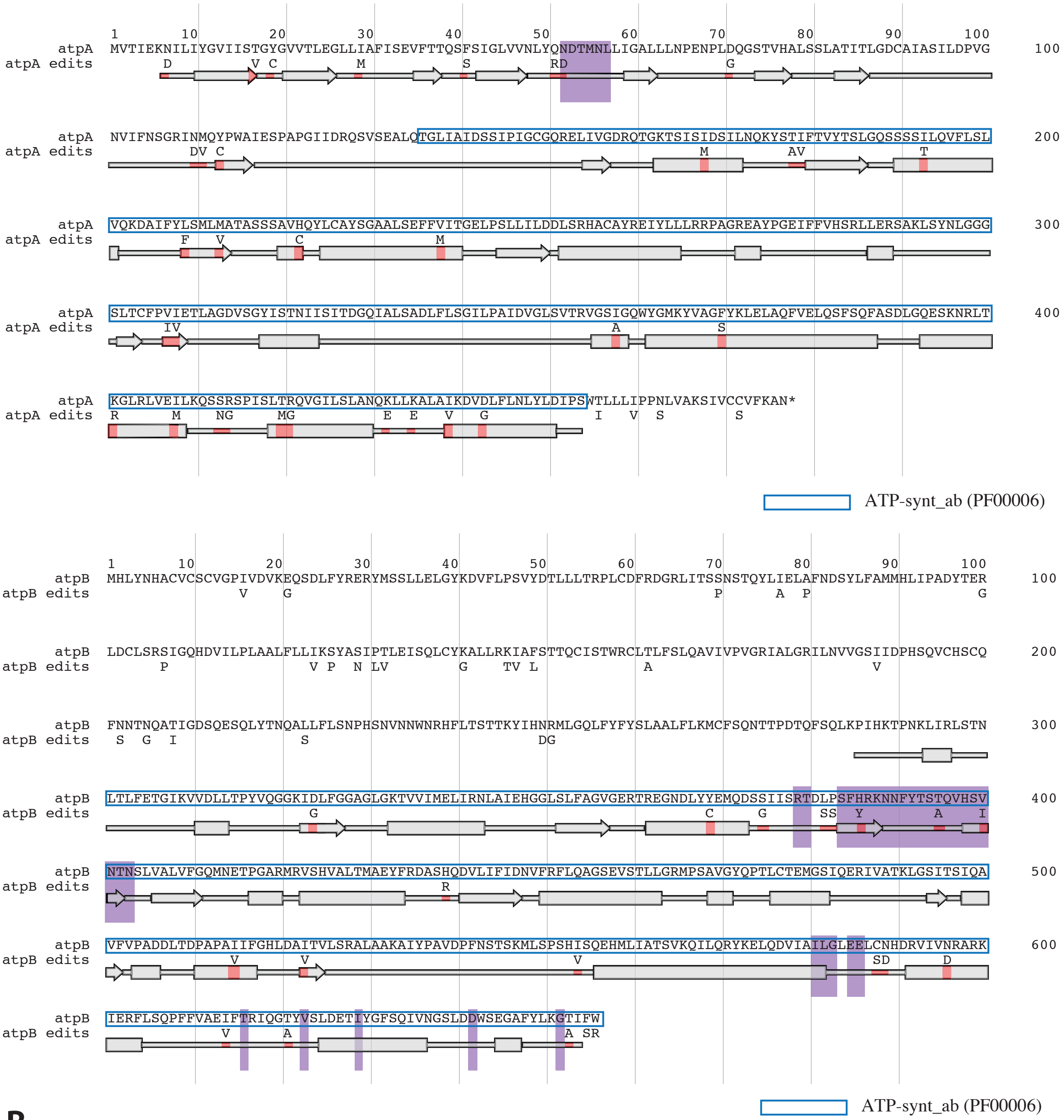
atpA
atpA edits
MVTIEKNILIYGVIISTGYGVVTLEGLLIAFISEVFTTQSFSIGLVVNLYQNDTMNLLIGALLLNPENPLDQGSTVHALSSLATITLGDCAIASILDPVG
NVIFNSGRINMQYPWAIESPAPGIIDRQSVSEALQTGLIAIDSSIPIGCGQRELIVGDRQTGKTSISIDSILNQKYSTIFTVYTSLGQSSSSILQVFLSL
VQKDAIFYLSMLMATASSSAVHQYLCAYSGAALSEFFVITGELPSLLILDDLSRHACAYREIYLLLRRPAGREAYPGEIFFVHSRLLERSAKLSYNLGGG
SLTCFPVIETLAGDVSGYISTNIISITDGQIALSADLFLSGILPAIDVGLSVTRVGSIGQWYGMKYVAGFYKLELAQFVELQSFSQFASDLGQESKNRLT
KGLRLVEILKQSSRSPISLTRQVGILSLANQKLLKALAIKDVDLFLNLYLDIPSWTLLLIPPNLVAKSIVCCVFKAN*
ATP-synt_ab (PF00006)
atpB
atpB edits
MHLYNHACVCSCVGPIVDVKEQSDLFYRERYMSSLLELGYKDVFLPSVYDTLLLTRPLCDFRDGRLITSSNSTQYLIELAFNDSYLFAMMHLIPADYTER
LDCLSRSIGQHDVILPLAALFLLIKSYASIPTLEISQLCYKALLRKIAFSTTQCISTWRCLTLFSLQAVIVPVGRIALGRILNVVGSIIDPHSQVCHSCQ
FNNTNQATIGDSQESQLYTNQALLFLSNPHSNVNNWNRHFLTSTTKYIHNRMLGQLFYFYSLAALFLKMCFSQNTTPDTQFSQLKPIHKTPNKLIRLSTN
LTLFETGIKVVDLLTPYVQGGKIDLFGGAGLGKTVVIMELIRNLAIEHGGLSLFAGVGERTREGNDLYYEMQDSSIISRTDLPSFHRKNNFYTSTQVHSV
NTNSLVALVFGQMNETPGARMRVSHVALTMAEYFRDASHQDVLIFIDNVFRFLQAGSEVSTLLGRMPSAVGYQPTLCTEMGSIQERIVATKLGSITSIQA
VFVPADDLTDPAPAIIFGHLDAITVLSRALAAKAIYPAVDPFNSTSKMLSPSHISQEHMLIATSVKQILQRYKELQDVIAILGLEELCNHDRVIVNRARK
IERFLSQPFFVAEIFTRIQGTYVSLDETIYGFSQIVNGSLDDWSEGAFYLKGTIFW
ATP-synt_ab (PF00006)

B

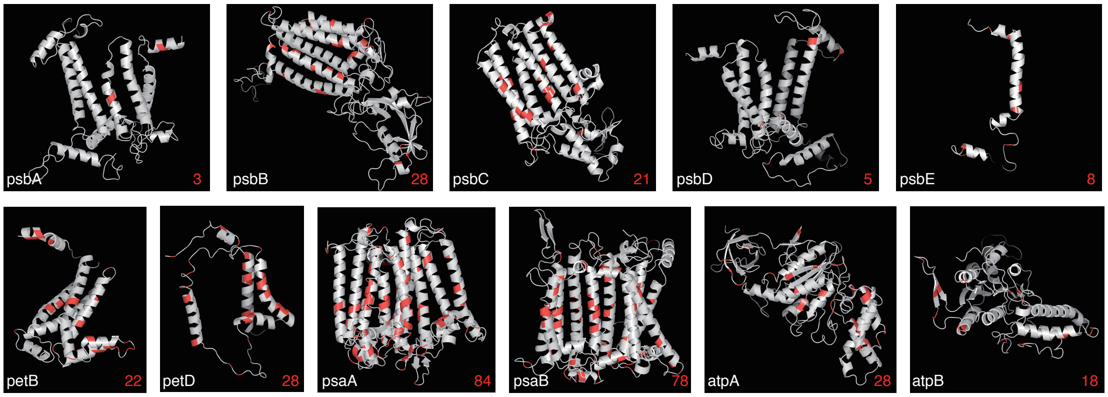
psbA 3
psbB 28
psbC 21
psbD 5
psbE 8
petB 22
petD 28
psaA 84
psaB 78
atpA 28
atpB 18

Supplementary Figure S6: Distribution of amino acid residues converted by RNA editing in plastid-encoded genes of *Symbiodinium minutum* and predicted secondary structures. (A) Lower case letters show edited amino acid residues. Conserved structures found by a pfam domain search are colored. Predicted secondary structures, helix (gray boxes), β-sheet (arrow boxes) and others (lines) are shown. Edited sites are marked in red. Regions showing significant conformational changes are highlighted in purple. **(B)** Secondary structures of plastid-encoded genes are also shown in 3D models. Residue numbers of amino acids converted by RNA editing are shown in red.
