# Supplementary material for: Massive Gene Transfer and Extensive RNA Editing of a Symbiotic Dinoflagellate Plastid Genome
Source: Genome Biol Evol. 2014 May 31;6(6):1408–22. doi: 10.1093/gbe/evu109 (PMC4079212; doi:10.1093/gbe/evu109)
Supplement: Supplementary Data [file supp_evu109_supplTable.pdf]

Table S1. List of plastid-associated genes including plastid-encoded, plastid-transferred and nuclear-transferred genes in *Symbiodinium minitum*.

Gene	No. after gene name	Gene model ID	Intron type			Transcriptome ID	scaffold	PFAM domain	KEGG ID
			GT-AG	GC-AG	GA-AG				
**Photosystem II**									
<u>*psbA*</u>		JX094319-20				symbB1.comp0_c0_seq1, mf105_rep_c1243	minicircle		K02703
<u>*psbB*</u>		JX094321-4				symbB1.comp28_c0_seq1, mf105_rep_c448	minicircle		K02704
<u>*psbC*</u>		JX094325-6				symbB1.comp52_c0_seq1, mf105_rep_c1265	minicircle		K02705
<u>*psbD*</u>		JX094327				symbB1.comp12_c0_seq1, mf105_rep_c2000	minicircle		K02706
<u>*psbE*</u>		JX094328-9				symbB1.comp2_c0_seq1, mf105_rep_c2000	minicircle		K02707
psbF	1	symbB.v1.2.037381	1	1	0	symbB1.EST_k37c20_49605	5492	Cytochrom_B559 (PF00283)	K02708
	2	symbB.v1.2.006949	7	4	1	symbB1.EST_k37c20_8605	416	Cytochrom_B559 (PF00283)	K02708
	3	symbB.v1.2.006950	3	4	1	symbB1.EST_k37c20_62297	416	Cytochrom_B559 (PF00283)	K02708
	4	symbB.v1.2.006948	4	4	0	symbB1.EST_k37c20_11166	416	Cytochrom_B559 (PF00283)	K02708
psbH	1	symbB.v1.2.035656	1	0	0	symbB1.EST_k37c20_14174	4856	PsbH (PF00737)	K02709
	2	symbB.v1.2.040833	0	0	0	symbB1.EST_k37c20_48001	7576	PsbH (PF00737)	K02709
	3	symbB.v1.2.013536	0	0	0	symbB1.EST_k37c20_7749	961	PsbH (PF00737)	K02709
	4	symbB.v1.2.013535	22	15	3	symbB1.EST_k37c20_45878	961	PsbH (PF00737)	K02709
	5	symbB.v1.2.013537	1	0	0	symbB1.EST_k37c20_45787	961	PsbH (PF00737)	K02709
	6	symbB.v1.2.013538	0	0	0	symbB1.EST_k37c20_61337	961	PsbH (PF00737)	K02709
<u>*psbI*</u>		JX094330				symbB1.comp1832_c0_seq1, mf105_c31159	minicircle		K02710
psbJ	1	symbB.v1.2.006330	2	0	0	symbB1.EST_k37c20_22776	378	PsbJ (PF01788)	
	2	symbB.v1.2.006331	0	0	0	–	378	PsbJ (PF01788)	
	3	symbB.v1.2.006332	14	9	5	symbB1.EST_k37c20_3665	378	PsbJ (PF01788)	
psbK		symbB.v1.2.040276	1	3	1	symbB1.EST_k37c20_12465	7118	PsbK (PF02533)	
psbL	1	symbB.v1.2.022100	8	5	2	symbB1.EST_k37c20_28278	1946	PsbL (PF02419)	K02713
	2	symbB.v1.2.034326	3	4	0	symbB1.EST_k37c20_15479	4412	PsbL (PF02419)	K02713
	3	symbB.v1.2.034330	1	3	1	–	4412	PsbL (PF02419)	K02713
	4	symbB.v1.2.034328	4	5	0	–	4412	PsbL (PF02419)	K02713
psbM	1	symbB.v1.2.011175	0	0	0	symbB1.EST_k37c20_19295	744	PsbM (PF05151)	K02714
	2	symbB.v1.2.011176	0	0	0	–	744	PsbM (PF05151)	
	3	symbB.v1.2.011177	0	0	0	symbB1.EST_k37c20_19294	744	PsbM (PF05151)	
	4	symbB.v1.2.011174	0	0	0	symbB1.EST_k37c20_19291	744	PsbM (PF05151)	
	5	symbB.v1.2.011173	0	0	0	–	744	PsbM (PF05151)	
	6	symbB.v1.2.039824	3	2	1	symbB1.EST_k37c20_59508	6809	PsbM (PF05151)	
psbN		symbB.v1.2.007196	11	15	1	symbB1.comp703_c0_seq1	436	PsbN (PF02468)	K02715
psbO	1	symbB.v1.2.023718	1	0	1	–	2193	MSP (PF01716)	K02716
	2	symbB.v1.2.013486	28	20	5	symbB1.EST_k37c20_5108, symbB1.EST_k37c20_10154, symbB1.EST_k37c20_2548, symbB1.EST_k37c20_2545	957	MSP (PF01716)	
	3	symbB.v1.2.023720	6	4	3	symbB1.EST_k37c20_43477, symbB1.EST_k37c20_43479, symbB1.EST_k37c20_56226, symbB1.EST_k37c20_2546, symbB1.EST_k37c20_43478	2193	MSP (PF01716)	
psbP	1	symbB.v1.2.031030	15	10	1	symbB1.EST_k37c20_14625	3559	PsbP (PF01789)	K02717
	2	symbB.v1.2.013147	8	13	1	symbB1.EST_k37c20_7108, symbB1.EST_k37c20_44209	925	PsbP (PF01789)	
psbQ		–				–		PsbQ (PF05757)	

psbR		–				–		PsbR (PF04725)	
psbS		–				–		–	
psbTc		–				–		PsbT (PF01405)	
psbTn		–				–		–	
psbU	1	symbB.v1.2.032533	9	6	7	symbB1.EST_k37c20_28312	3918	PsbU (PF06514), Myb_DNA-bind_6 (PF13921)	K02719
	2	symbB.v1.2.032534	2	0	0	symbB1.EST_k37c20_1039	3918	PsbU (PF06514), PsbU (PF06514), PsbU (PF06514)	
psbV	1	symbB.v1.2.040053	2	0	1	symbB1.EST_k37c20_62309	6958	Cytochrom_C (PF00034)	K02720
	2	symbB.v1.2.041562	2	0	1	symbB1.EST_k37c20_55685	8355	Cytochrom_C (PF00034)	
	3	symbB.v1.2.041561	1	0	0	–	8355	Cytochrom_C (PF00034)	
	4	symbB.v1.2.040054	2	0	1	symbB1.EST_k37c20_48299	6958	Cytochrome_CBB3 (PF13442)	
psbW		–				–		PsbW (PF07123)	K08903
psbX		–				–		PsbX (PF06596)	
psbY	1	symbB.v1.2.029162	1	0	0	symbB1.EST_k37c20_471	3163	PsbY (PF06298)	K02723
	2	symbB.v1.2.034824	3	3	0	symbB1.EST_k37c20_8760	456	PsbY (PF06298)	
	3	symbB.v1.2.038177	3	1	0	symbB1.EST_k37c20_19997	5861	PsbY (PF06298)	
psbZ		–				–		Ycf9 (PF01737)	K02724
psb27		symbB.v1.2.001675	1	10	1	symbB1.EST_k37c20_10935	86	PSII_Pbs27 (PF13326)	K08902
psb28		–				–		Psb28 (PF03912)	
psb29		symbB.v1.2.025262	7	12	1	symbB1.EST_k37c20_6259	2444	ThylakoidFormat (PF11264)	
psb30/ycf 12		symbB.v1.2.019436	0	0	0	symbB1.EST_k37c20_11334	1590	PSII_Ycf12 (PF05969)	
psb31		–				–		–	
lhcb	1	symbB.v1.2.000416	8	7	8	symbB1.comp258_c0_seq2	30	Chloroa_b-bind (PF00504)	
	2	symbB.v1.2.000417	8	7	9	symbB1.EST_k37c20_6890	30	Chloroa_b-bind (PF00504)	
	3	symbB.v1.2.000704	10	10	3	symbB1.EST_k37c20_1429	40	Chloroa_b-bind (PF00504)	
	4	symbB.v1.2.002584	1	0	0	symbB1.EST_k37c20_19074	135	Chloroa_b-bind (PF00504)	
	5	symbB.v1.2.002585	8	0	1	symbB1.EST_k37c20_17230	135	Chloroa_b-bind (PF00504)	
	6	symbB.v1.2.003713	13	8	6	symbB1.comp15162_c0_seq1, symbB1.comp23584_c0_seq1, symbB1.comp16437_c0_seq1	209	Chloroa_b-bind (PF00504)	
	7	symbB.v1.2.003714	1	2	0	symbB1.EST_k37c20_11015	209	Chloroa_b-bind (PF00504)	
	8	symbB.v1.2.003715	33	11	10	symbB1.EST_k37c20_11024, symbB1.EST_k37c20_11028	209	Chloroa_b-bind (PF00504)	
	9	symbB.v1.2.004087	10	11	2	symbB1.EST_k37c20_21946	200	Chloroa_b-bind (PF00504)	
	10	symbB.v1.2.004366	7	8	2	symbB1.EST_k37c20_2750	240	Chloroa_b-bind (PF00504)	
	11	symbB.v1.2.005126	6	6	1	symbB1.EST_k37c20_1821, symbB1.EST_k37c20_1820	226	Chloroa_b-bind (PF00504), MIP (PF00230)	
	12	symbB.v1.2.005231	40	29	14	symbB1.EST_k37c20_4845, symbB1.EST_k37c20_4846, symbB1.EST_k37c20_11532	293	Epimerase (PF01370), cNMP_binding (PF00027), Chloroa_b-bind (PF00504)	
	13	symbB.v1.2.005251	7	6	1	symbB1.EST_k37c20_7127	304	Chloroa_b-bind (PF00504)	

14	symbB.v1.2.005437	12	8	4	symbB1.EST_k37c20_6587	312	Chloroa_b-bind (PF00504)
15	symbB.v1.2.006212	23	16	12	symbB1.EST_k37c20_8481, symbB1.EST_k37c20_877	355	Chloroa_b-bind (PF00504)
16	symbB.v1.2.006213	20	18	8	symbB1.EST_k37c20_2795	355	Chloroa_b-bind (PF00504)
17	symbB.v1.2.006472	9	8	3	symbB1.EST_k37c20_32759, symbB1.EST_k37c20_7255	325	Epimerase (PF01370), cNMP_binding (PF00027), Chloroa_b-bind (PF00504)
18	symbB.v1.2.006473	6	15	4	symbB1.EST_k37c20_7255, symbB1.EST_k37c20_4302	325	Chloroa_b-bind (PF00504)
19	symbB.v1.2.006873	3	6	1	symbB1.EST_k37c20_644	382	Chloroa_b-bind (PF00504)
20	symbB.v1.2.006875	16	13	2	symbB1.EST_k37c20_14960, symbB1.EST_k37c20_14962, symbB1.EST_k37c20_2817, symbB1.EST_k37c20_2820	382	Chloroa_b-bind (PF00504)
21	symbB.v1.2.007477	7	7	3	symbB1.EST_k37c20_47102, symbB1.EST_k37c20_47103	410	Chloroa_b-bind (PF00504)
22	symbB.v1.2.007571	11	6	1	symbB1.EST_k37c20_24441	461	Chloroa_b-bind (PF00504)
23	symbB.v1.2.007877	8	6	2	symbB1.EST_k37c20_16591	482	Chloroa_b-bind (PF00504)
24	symbB.v1.2.008549	2	2	0	symbB1.EST_k37c20_20167	538	
25	symbB.v1.2.010108	19	13	8	symbB1.EST_k37c20_16307, symbB1.EST_k37c20_16308, symbB1.EST_k37c20_2507	654	Chloroa_b-bind (PF00504)
26	symbB.v1.2.010109	21	14	4	symbB1.EST_k37c20_475	654	Chloroa_b-bind (PF00504)
27	symbB.v1.2.010521	17	9	1	symbB1.EST_k37c20_12044, symbB1.EST_k37c20_12045, symbB1.EST_k37c20_12043, symbB1.EST_k37c20_4242	690	Chloroa_b-bind (PF00504)
28	symbB.v1.2.010522	19	8	4	symbB1.EST_k37c20_31535	690	Chloroa_b-bind (PF00504), dDENN (PF03455), DUF547 (PF04784)
29	symbB.v1.2.011071	10	9	0	symbB1.EST_k37c20_2584, symbB1.EST_k37c20_2583	738	Chloroa_b-bind (PF00504)
30	symbB.v1.2.011270	1	1	1	symbB1.EST_k37c20_38079	753	Chloroa_b-bind (PF00504)
31	symbB.v1.2.011302	2	1	1	symbB1.EST_k37c20_47037, symbB1.EST_k37c20_7606	735	Chloroa_b-bind (PF00504)
32	symbB.v1.2.011361	28	16	15	symbB1.EST_k37c20_7019, symbB1.EST_k37c20_48349, symbB1.EST_k37c20_23874	741	Chloroa_b-bind (PF00504)
33	symbB.v1.2.012424	2	0	1	symbB1.EST_k37c20_4579, symbB1.EST_k37c20_59568	849	Chloroa_b-bind (PF00504)
34	symbB.v1.2.012918	20	17	8	symbB1.EST_k37c20_13288, symbB1.EST_k37c20_36454	903	FtsJ (PF01728), Chloroa_b-bind (PF00504)
35	symbB.v1.2.016027	12	8	7	symbB1.EST_k37c20_10495, symbB1.EST_k37c20_20836	1188	Chloroa_b-bind (PF00504)
36	symbB.v1.2.016170	3	1	0	–	1226	Chloroa_b-bind (PF00504)
37	symbB.v1.2.016171	3	3	0	–	1226	Chloroa_b-bind (PF00504)
38	symbB.v1.2.016253	3	3	1	symbB1.EST_k37c20_3009	1233	Chloroa_b-bind (PF00504)
39	symbB.v1.2.016255	8	6	2	symbB1.EST_k37c20_4037	1233	Chloroa_b-bind (PF00504)

40	symbB.v1.2.016256	0	0	0	symbB1.EST_k37c20_4035, symbB1.EST_k37c20_4036, symbB1.EST_k37c20_4038	1233	Chloroa_b-bind (PF00504)
41	symbB.v1.2.016257	1	1	4	symbB1.EST_k37c20_4035, symbB1.EST_k37c20_4036, symbB1.EST_k37c20_4038	1233	Chloroa_b-bind (PF00504)
42	symbB.v1.2.016373	4	1	1	symbB1.EST_k37c20_25398	1243	Chloroa_b-bind (PF00504)
43	symbB.v1.2.017478	12	6	10	symbB1.EST_k37c20_17472	1363	Chloroa_b-bind (PF00504)
44	symbB.v1.2.017479	6	0	1	–	1363	Chloroa_b-bind (PF00504)
45	symbB.v1.2.017480	18	11	8	symbB1.EST_k37c20_17467	1363	Chloroa_b-bind (PF00504)
46	symbB.v1.2.018027	16	10	7	symbB1.EST_k37c20_49160	1374	Chloroa_b-bind (PF00504)
47	symbB.v1.2.019686	18	7	10	symbB1.EST_k37c20_3155	1622	Chloroa_b-bind (PF00504)
48	symbB.v1.2.019932	7	3	2	–	1652	Exo_endo_phos (PF03372)
49	symbB.v1.2.019935	0	0	0	–	1652	Chloroa_b-bind (PF00504)
50	symbB.v1.2.020974	3	13	1	symbB1.EST_k37c20_4529	1791	Chloroa_b-bind (PF00504)
51	symbB.v1.2.022533	12	9	1	–	2006	LRR_5 (PF13306), Chloroa_b-bind (PF00504)
52	symbB.v1.2.023630	0	0	0	–	2176	Chloroa_b-bind (PF00504)
53	symbB.v1.2.024680	6	11	3	symbB1.EST_k37c20_14787	2350	Chloroa_b-bind (PF00504)
54	symbB.v1.2.024681	7	9	4	symbB1.EST_k37c20_14788	2350	Chloroa_b-bind (PF00504)
55	symbB.v1.2.024854	5	4	3	–	2380	Chloroa_b-bind (PF00504)
56	symbB.v1.2.024892	17	13	4	symbB1.EST_k37c20_76, symbB1.EST_k37c20_43447	2388	Nucleotid_trans (PF03407), Chloroa_b-bind (PF00504)
57	symbB.v1.2.026613	10	13	3	symbB1.EST_k37c20_1949	2674	Chloroa_b-bind (PF00504)
58	symbB.v1.2.026778	14	7	2	symbB1.EST_k37c20_32042, symbB1.EST_k37c20_58129	2704	Chloroa_b-bind (PF00504)
59	symbB.v1.2.028203	5	4	9	symbB1.EST_k37c20_36237	2965	Chloroa_b-bind (PF00504)
60	symbB.v1.2.028302	0	0	0	–	2947	Chloroa_b-bind (PF00504)
61	symbB.v1.2.028303	8	0	1	–	2947	Chloroa_b-bind (PF00504)
62	symbB.v1.2.028352	7	8	3	symbB1.EST_k37c20_4560	2996	Chloroa_b-bind (PF00504), Coprogen_oxidas (PF01218)
63	symbB.v1.2.028353	22	21	10	symbB1.EST_k37c20_28632	2996	Chloroa_b-bind (PF00504), Exostosin (PF03016)
64	symbB.v1.2.028830	9	10	0	symbB1.EST_k37c20_36093	3090	Chloroa_b-bind (PF00504)
65	symbB.v1.2.029304	0	0	0	symbB1.EST_k37c20_3323, symbB1.EST_k37c20_3324	3191	Chloroa_b-bind (PF00504)
66	symbB.v1.2.029305	0	0	0	–	3191	Chloroa_b-bind (PF00504)
67	symbB.v1.2.030315	9	5	1	symbB1.EST_k37c20_627, symbB1.EST_k37c20_4455, symbB1.EST_k37c20_55765	3402	Chloroa_b-bind (PF00504), Rieske (PF00355)
68	symbB.v1.2.030436	5	6	3	symbB1.EST_k37c20_13237	3430	Chloroa_b-bind (PF00504)

69	symbB.v1.2.030463	2	2	0	symbB1.EST_k37c20_41411	3437	Chloroa_b-bind (PF00504), ubiquitin (PF00240)
70	symbB.v1.2.030607	4	4	1	–	3404	Chloroa_b-bind (PF00504)
71	symbB.v1.2.030608	6	6	1	symbB1.EST_k37c20_1458	3404	Chloroa_b-bind (PF00504)
72	symbB.v1.2.030609	6	2	2	symbB1.EST_k37c20_1460, symbB1.EST_k37c20_1456, symbB1.EST_k37c20_1455, symbB1.EST_k37c20_1458	3404	Chloroa_b-bind (PF00504)
73	symbB.v1.2.033058	25	23	7	symbB1.EST_k37c20_5361, symbB1.EST_k37c20_7743	4047	Peptidase_M16_C (PF05193), Chloroa_b-bind (PF00504)
74	symbB.v1.2.033169	6	1	0	symbB1.EST_k37c20_14407	4084	Chloroa_b-bind (PF00504)
75	symbB.v1.2.033532	18	5	2	–	4179	La (PF05383), Chloroa_b-bind (PF00504)
76	symbB.v1.2.033682	3	1	3	symbB1.EST_k37c20_9962, symbB1.EST_k37c20_9963, symbB1.EST_k37c20_9964	4219	Chloroa_b-bind (PF00504)
77	symbB.v1.2.034225	9	9	3	–	4384	Chloroa_b-bind (PF00504)
78	symbB.v1.2.037219	4	3	0	symbB1.EST_k37c20_2818	5435	Chloroa_b-bind (PF00504)
79	symbB.v1.2.037220	13	4	1	symbB1.EST_k37c20_10475	5435	Chloroa_b-bind (PF00504)
80	symbB.v1.2.037221	3	3	0	–	5435	Chloroa_b-bind (PF00504)
81	symbB.v1.2.037222	1	0	0	symbB1.EST_k37c20_2818	5435	Chloroa_b-bind (PF00504)
82	symbB.v1.2.037223	7	1	0	symbB1.EST_k37c20_5038	5435	Chloroa_b-bind (PF00504)
83	symbB.v1.2.037312	0	0	0	–	5475	Chloroa_b-bind (PF00504)
84	symbB.v1.2.037313	1	0	1	–	5475	Chloroa_b-bind (PF00504)
85	symbB.v1.2.037730	0	0	0	symbB1.EST_k37c20_3367	5651	Chloroa_b-bind (PF00504)
86	symbB.v1.2.038122	0	0	0	–	5832	Chloroa_b-bind (PF00504)
87	symbB.v1.2.038123	0	0	0	–	5832	Chloroa_b-bind (PF00504)
88	symbB.v1.2.038124	23	24	18	symbB1.EST_k37c20_28902, symbB1.EST_k37c20_55802	5832	Chloroa_b-bind (PF00504)
89	symbB.v1.2.038157	15	4	3	symbB1.EST_k37c20_642, symbB1.EST_k37c20_643, symbB1.EST_k37c20_645	5850	Chloroa_b-bind (PF00504), RVT_2 (PF07727)
90	symbB.v1.2.038449	9	8	10	symbB1.EST_k37c20_8977	5990	Chloroa_b-bind (PF00504)
91	symbB.v1.2.038694	3	1	2	–	6128	Chloroa_b-bind (PF00504)
92	symbB.v1.2.039010	6	2	1	–	6293	Chloroa_b-bind (PF00504)
93	symbB.v1.2.039011	4	3	1	–	6293	Chloroa_b-bind (PF00504)
94	symbB.v1.2.039815	10	5	1	–	6803	Chloroa_b-bind (PF00504)
95	symbB.v1.2.041094	8	0	0	–	7819	Chloroa_b-bind (PF00504)
96	symbB.v1.2.041290	1	0	0	–	8023	Chloroa_b-bind (PF00504)
97	symbB.v1.2.041291	9	6	4	–	8023	Chloroa_b-bind (PF00504)
98	symbB.v1.2.042135	2	1	1	–	9320	Chloroa_b-bind (PF00504)

	99	symbB.v1.2.042413	2	0	0	–	8178	Chloroa_b-bind (PF00504)	
	100	symbB.v1.2.042811	2	0	0	–	11148	Chloroa_b-bind (PF00504)	
	101	symbB.v1.2.043368	1	0	0	–	14023	Chloroa_b-bind (PF00504)	
**Photosystem I**									
psaA		JX094315-7				symbB1.comp56_c0_seq1, mf105_rep_c3118, mf105_rep_c3002	minicircle		K02689
psaB		JX094318				symbB1.comp37_c0_seq1, mf105_rep_c1527	minicircle		K02690
psaC		symbB.v1.2.013207	3	4	1	symbB1.EST_k37c20_56198	929	Fer4_7 (PF12838)	K02691
psaD	1	symbB.v1.2.037582	0	0	0	symbB1.EST_k37c20_6446	5592	PsaD (PF02531)	K02692
	2	symbB.v1.2.039732	11	12	1	symbB1.EST_k37c20_22702, symbB1.EST_k37c20_49742	6754	PsaD (PF02531)	K02692
	3	symbB.v1.2.037583	0	0	0	symbB1.EST_k37c20_59567, symbB1.EST_k37c20_6446, symbB1.EST_k37c20_2434	5592	PsaD (PF02531)	K02692
psaE	1	symbB.v1.2.001643	5	3	0	symbB1.EST_k37c20_55546, symbB1.EST_k37c20_3550	86	PSI_PsaE (PF02427)	K02693
	2	symbB.v1.2.028843	1	1	0	symbB1.EST_k37c20_9048	3093	PSI_PsaE (PF02427)	K02693
psaF	1	symbB.v1.2.025465	2	0	0	–	2472	PSI_PsaF (PF02507)	K02694
	2	symbB.v1.2.025464	4	2	0	symbB1.EST_k37c20_8188, symbB1.EST_k37c20_1974	2472	PSI_PsaF (PF02507)	K02694
	3	symbB.v1.2.012847	2	0	0	symbB1.EST_k37c20_44666	887	PSI_PsaF (PF02507)	K02694
psaG		–				–		PSI_PSAK (PF01241)	
psaH		–				–		PSI_PsaH (PF03244)	
psaI		–				–		PSI_8 (PF00796)	
psaJ	1	symbB.v1.2.027533	5	4	0	symbB1.EST_k37c20_2543	2832	PSI_PsaJ (PF01701)	
	2	symbB.v1.2.027534	5	4	0	symbB1.EST_k37c20_21955	2832	PSI_PsaJ (PF01701)	
	3	symbB.v1.2.027535	16	24	13	symbB1.EST_k37c20_13143	2832	PSI_PsaJ (PF01701)	
psaK		–				–		PSI_PSAK (PF01241)	
psaL	1	symbB.v1.2.037928	3	0	0	symbB1.EST_k37c20_2082	5742	PsaL (PF02605)	
	2	symbB.v1.2.037929	1	0	0	symbB1.EST_k37c20_16360	5742	PsaL (PF02605)	
	3	symbB.v1.2.027137	7	4	4	symbB1.EST_k37c20_8521	2764	PsaL (PF02605)	
psaM		–				–		PsaM (PF07465)	
psaN		–				–		PsaN (PF05479)	
psaO		–				–		–	
psaP		–				–		DUF4308 (PF14159)	
psaX		–				–		PsaX (PF08078)	
lhca		–				–		Chloroa_b-bind (PF00504)	
**Cytochrome b6f complex**									
petA	1	symbB.v1.2.018517	15	3	6	symbB1.EST_k37c20_4757, symbB1.EST_k37c20_4769, symbB1.EST_k37c20_4770	1480	Apocytochr_F_C (PF01333)	K02634
	2	symbB.v1.2.018518	6	1	3	symbB1.EST_k37c20_4765, symbB1.EST_k37c20_4752, symbB1.EST_k37c20_4767	1480	Apocytochr_F_C (PF01333)	
	3	symbB.v1.2.018520	5	4	3	symbB1.EST_k37c20_8370, symbB1.EST_k37c20_4763, symbB1.EST_k37c20_4782	1480	Apocytochr_F_C (PF01333)	

	4	symbB.v1.2.020196	3	1	2	symbB1.EST_k37c20_4777	1681	Apocytochr_F_C (PF01333)	
	5	symbB.v1.2.020193	12	9	2	symbB1.EST_k37c20_4778	1681	Apocytochr_F_C (PF01333)	
petB		JX094309-10				symbB1.EST_k37c20_1147, mf105_rep_c1660	minicircle		K02635
petC	1	symbB.v1.2.006097	19	10	6	symbB1.EST_k37c20_2827	359	CytB6-F_Fe-S (PF08802), Rieske (PF00355)	K02636
	2	symbB.v1.2.006095	3	0	0	symbB1.EST_k37c20_11748	359	CytB6-F_Fe-S (PF08802), Rieske (PF00355)	
	3	symbB.v1.2.006093	2	1	0	symbB1.EST_k37c20_11749	359	CytB6-F_Fe-S (PF08802), Rieske (PF00355)	
	4	symbB.v1.2.006094	2	1	0	symbB1.EST_k37c20_11747	359	CytB6-F_Fe-S (PF08802), Rieske (PF00355)	
petD		JX094311-14				symbB1.comp26_c0_seq1, mf105_rep_c797	minicircle		K02637
petG	1	symbB.v1.2.003308	5	7	4	symbB1.EST_k37c20_32837	182	PetG (PF02529)	
	2	symbB.v1.2.000994	11	4	0	symbB1.EST_k37c20_17957	43	PetG (PF02529)	
	3	symbB.v1.2.009131	3	1	0	symbB1.EST_k37c20_56678	577	PetG (PF02529)	
petL		–				–		PetL (PF05115)	
petM		–				–		PetM (PF08041)	
petN	1	symbB.v1.2.029346	0	0	0	symbB1.EST_k37c20_48119	3201	PetN (PF03742)	
	2	symbB.v1.2.029345	0	0	0	symbB1.EST_k37c20_48119	3201	PetN (PF03742)	
petO		–				–			
ccsA		symbB.v1.2.004806	12	14	3		270	Cytochrom_C_asm (PF01578)	
ccs1		symbB.v1.2.008118	8	6	1	symbB1.EST_k37c20_20096	500	ResB (PF05140)	K07399
**Cytochrome c6**									
petJ	1	symbB.v1.2.004909	5	3	0	symbB1.EST_k37c20_31635	263	Cytochrome_CBB3 (PF13442), Cytochrom_C (PF00034)	K08906
	2	symbB.v1.2.031786	0	0	0	symbB1.EST_k37c20_64213, symbB1.EST_k37c20_5653, symbB1.EST_k37c20_55768	3728	Cytochrome_CBB3 (PF13442)	K08906
	3	symbB.v1.2.036874	0	0	0	symbB1.EST_k37c20_5651, symbB1.EST_k37c20_55769,symbB1.EST_k37c20_5650	5310	Cytochrome_CBB3 (PF13442)	K08906
petF	1	symbB.v1.2.035732	0	0	0	-	4875	Fer2 (PF00111)	K02639
	2	symbB.v1.2.035731	0	0	0	symbB1.EST_k37c20_1362, symbB1.EST_k37c20_1364, symbB1.EST_k37c20_1361, symbB1.EST_k37c20_1360	4875	Fer2 (PF00111)	K02639
	3	symbB.v1.2.037890	0	0	0	symbB1.EST_k37c20_44125, symbB1.EST_k37c20_44126, symbB1.EST_k37c20_1363	5725	Fer2 (PF00111)	K02639
petH	1	symbB.v1.2.040184	2	2	0	symbB1.EST_k37c20_58858, symbB1.EST_k37c20_5214	7056	NAD_binding_1 (PF00175)	K02641
	2	symbB.v1.2.013928	4	2	1	symbB1.EST_k37c20_149	996	NAD_binding_1 (PF00175)	
	3	symbB.v1.2.014541	12	3	4	symbB1.EST_k37c20_11159	1066	NAD_binding_1 (PF00175), Lipase_3 (PF01764)	
	4	symbB.v1.2.014542	2	3	3	symbB1.EST_k37c20_11157, symbB1.EST_k37c20_11158	1066	NAD_binding_1 (PF00175)	
	5	symbB.v1.2.036979	4	5	2	symbB1.EST_k37c20_46801	5347	NAD_binding_1 (PF00175)	

	6	symbB.v1.2.035012	23	15	8	symbB1.EST_k37c20_4294	4627	DUF4326 (PF14216), Exostosin (PF03016), NAD_binding_1 (PF00175)	
ftrC		symbB.v1.2.003535	16	12	8	symbB1.EST_k37c20_24201	196	FeThRed_B (PF02943)	K00535
trxM		symbB.v1.2.031355	0	0	0	symbB1.EST_k37c20_60599	3632	Thioredoxin (PF00085)	K03671
**ATP synthase**									
atpA		JX094304-6				symbB1.comp193_c0_seq2, mf105_rep_c2696	minicircle		K02111
atpB		JX094307-8				symbB1.comp144_c0_seq1, mf105_rep_c562	minicircle		K02112
atpC	1	symbB.v1.2.004028	6	4	3	symbB1.EST_k37c20_7574	227	ATP-synt (PF00231)	
	2	symbB.v1.2.004029	6	4	3	symbB1.EST_k37c20_7572, symbB1.EST_k37c20_7574	227	ATP-synt (PF00231)	
	3	symbB.v1.2.004031	12	11	4	symbB1.EST_k37c20_7575, symbB1.EST_k37c20_29651,symbB1.EST_k37c20_46079	227	ATP-synt (PF00231)	
atpD	1	symbB.v1.2.020826	1	1	2	symbB1.EST_k37c20_7487	1727	OSCP (PF00213)	K02113/0
	2	symbB.v1.2.030497	1	0	0	symbB1.EST_k37c20_16347	3435	OSCP (PF00213)	K02113/0
	3	symbB.v1.2.030498	0	0	0	symbB1.EST_k37c20_16348	3435	OSCP (PF00213)	K02113/0
atpE	1	symbB.v1.2.033719	11	7	2	symbB1.EST_k37c20_47825, symbB1.EST_k37c20_22113, symbB1.EST_k37c20_18318	4230	ATP-synt_DE_N (PF02823)	
	2	symbB.v1.2.013177	3	3	3	symbB1.EST_k37c20_46231, symbB1.EST_k37c20_29422	926	ATP-synt_DE_N (PF02823)	K02114
atpF	1	symbB.v1.2.029247	15	14	8	symbB1.comp1997_c0_seq1	3178	ATP-synt_B (PF00430)	
	2	symbB.v1.2.001381	8	9	0	symbB1.comp36530_c0_seq2	67	ATP-synt_B (PF00430)	
	3	symbB.v1.2.022253	11	9	4	symbB1.comp1138_c0_seq1	1966	ATP-synt_B (PF00430)	
atpG	1	symbB.v1.2.007301	4	2	3	symbB1.EST_k37c20_46210	440	ATP-synt_B (PF00430)	
	2	symbB.v1.2.007300	4	3	2	symbB1.EST_k37c20_25042	440	ATP-synt_B (PF00430)	
atpH	1	symbB.v1.2.000251	0	0	0	symbB1.EST_k37c20_22098	18	ATP-synt_C (PF00137)	K02110
	2	symbB.v1.2.011430	0	0	0	symbB1.EST_k37c20_8674	767	ATP-synt_C (PF00137)	K02110
	3	symbB.v1.2.003777	10	11	7	symbB1.EST_k37c20_9469	207	ATP-synt_C (PF00137)	K02110
atpI	1	symbB.v1.2.032748	5	6	1	symbB1.EST_k37c20_1788	3968	ATP-synt_A (PF00119)	K02108
	2	symbB.v1.2.032747	6	5	1	symbB1.EST_k37c20_1787	3968	ATP-synt_A (PF00119)	K02108
**Phycobiliproteins**									
apcA		–				–		Phycobilisome (PF00502)	K02092
apcB		–				–		Phycobilisome (PF00502)	K02093
apcD		–				–		Phycobilisome (PF00502)	K02095
apcE		–				–		Phycobilisome (PF00502)	K02096
apcF		–				–		Phycobilisome (PF00502)	K02097
cpcA		–				–		Phycobilisome (PF00502)	K02284

cpcB		–				–		Phycobilisome (PF00502)	K02285
**Metabolism**									
**Carbohydrates**									
cfxQ		symbB.v1.2.028709	29	25	9	symbB1.EST_k37c20_14378	3062	AAA (PF00004)	K06413
rbcL		symbB.v1.2.041360	3	0	0	symbB1.EST_k37c20_13147	8099	RuBisCO_large (PF00016)	K01601
**Lipids**									
acpP	1	symbB.v1.2.020352	5	1	2	symbB1.EST_k37c20_46799	1703	PP-binding (PF00550)	K02078
	2	symbB.v1.2.020351	23	12	3	symbB1.EST_k37c20_25813	1703	PP-binding (PF00550)	
**Nucleotides**									
carA		symbB.v1.2.027619	3	5	3	symbB1.EST_k37c20_3378, symbB1.EST_k37c20_9006	2810	CPSase_sm_chain (PF00988)	K01956/0
**Amino acids**									
gltB	1	symbB.v1.2.014989	45	37	20	symbB1.EST_k37c20_6414	1088	GATase_2 (PF00310)	K00264
	2	symbB.v1.2.020534	18	14	5	symbB1.EST_k37c20_22583	1735	GATase_2 (PF00310)	
	3	symbB.v1.2.017142	30	25	12	symbB1.EST_k37c20_469	1327	GATase_2 (PF00310)	
ilvB		symbB.v1.2.032800	18	14	2	symbB1.EST_k37c20_3325	3984	TPP_enzyme_N (PF02776)	K01652
**Complex sugars**									
glmS	1	symbB.v1.2.007867	13	9	2	symbB1.EST_k37c20_19261	448	SIS (PF01380)	K00820
	2	symbB.v1.2.024317	27	31	11	symbB1.EST_k37c20_17650	2282	SIS (PF01380)	K00820
**Cofactors**									
chlI	1	symbB.v1.2.025433	12	13	3	symbB1.EST_k37c20_7018	2466	Mg_chelatase (PF01078)	K03405
	2	symbB.v1.2.031914	7	0	0	symbB1.EST_k37c20_13445	3736	Mg_chelatase (PF01078)	K03405
cobA		symbB.v1.2.015591	8	8	2	symbB1.EST_k37c20_50526	1127	TP_methylase (PF00590)	K02303
preA		symbB.v1.2.008416	6	14	2	symbB1.EST_k37c20_51767	529	polyprenyl_synt (PF00348)	K05356
**Transport**									
secA	1	symbB.v1.2.013318	0	0	0	symbB1.EST_k37c20_32791	938	SecA_DEAD (PF07517)	K03070
	2	symbB.v1.2.028305	15	20	14	symbB1.EST_k37c20_3645	2947	SecA_DEAD (PF07517)	K03070
	3	symbB.v1.2.024712	13	10	2	symbB1.EST_k37c20_20304	2359	SecA_DEAD (PF07517)	
secY		symbB.v1.2.012783	15	15	5	symbB1.EST_k37c20_19185, symbB1.EST_k37c20_33447	859	SecY (PF00344)	K03076
sufB		symbB.v1.2.011807	27	35	18	symbB1.EST_k37c20_9193, symbB1.EST_k37c20_9481	773	UPF0051 (PF01458)	K09014
tatC		symbB.v1.2.030075	33	10	3	symbB1.EST_k37c20_39615	3344	TatC (PF00902)	K03118
**Genetic system**									
**RNA polymerase**									
rpoA		symbB1.v1.2.001673	21	14	15	symbB1.EST_k37c20_35742	86	RNA_pol_A_CTD (PF03118)	K03040/0
rpoB	1	symbB.v1.2.028322	23	17	6	symbB1.EST_k37c20_13857	2993	RNA_pol_Rpb2_6 (PF00562)	K03043
	2	symbB.v1.2.028323	15	13	4	symbB1.EST_k37c20_13857	2993	RNA_pol_Rpb2_3 (PF04565)	
rpoC1		symbB.v1.2.030589	11	11	4	symbB1.EST_k37c20_7300	3465	RNA_pol_Rpb1_2 (PF00623)	K03046
rpoC2		symbB.v1.2.011942	43	32	16	symbB1.EST_k37c20_22007 , symbB1.EST_k37c20_37665	814	RNA_pol_Rpb1_5 (PF04998), RNA_pol_Rpb1_4 (PF05000)	K03046
**Translation**									

infB		symbB.v1.2.007711	22	39	21	symbB1.EST_k37c20_3131, symbB1.EST_k37c20_23525, symbB1.EST_k37c20_12845	479	IF2_N (PF04760)	K02519
tsf		symbB.v1.2.029333	5	7	7	symbB1.EST_k37c20_13647	3199	EF_TS (PF00889)	K02357
tufA		symbB.v1.2.025951	5	11	3	symbB1.EST_k37c20_2835, symbB1.EST_k37c20_2836, symbB1.EST_k37c20_2837	2552	GTP_EFTU (PF00009, PF03144, PF03143)	K03231
**Ribosomal Proteins**									
rpl1		symbB.v1.2.009383	17	10	3	symbB1.EST_k37c20_11396	595	Ribosomal_L1 (PF00687)	K02863
rpl2		symbB.v1.2.007444	28	16	9	symbB1.EST_k37c20_47934, symbB1.EST_k37c20_36558, symbB1.EST_k37c20_31287	445	Ribosomal_L2_C (PF03947)	K02886
rpl3		symbB.v1.2.003502	7	7	5	symbB1.EST_k37c20_48217	197	Ribosomal_L3 (PF00297)	K02906
rpl3		symbB.v1.2.020107	11	6	4	symbB1.EST_k37c20_30891	1674	Ribosomal_L3 (PF00297)	K02906
rpl11	1	symbB.v1.2.001567	3	13	1	symbB1.EST_k37c20_27089	3577	Ribosomal_L11_N (PF03946)	K02867
	2	symbB.v1.2.031116	0	0	0	symbB1.EST_k37c20_46642	88	Ribosomal_L11_N (PF03946)	K02867
rpl12	1	symbB.v1.2.006936	10	4	4	symbB1.EST_k37c20_35887	400	Ribosomal_L12 (PF00542)	K02935
	2	symbB.v1.2.007734	10	9	4	symbB1.EST_k37c20_43211	457	Ribosomal_L12 (PF00542)	K02935
	3	symbB.v1.2.016557	10	8	4	symbB1.EST_k37c20_5665	1261	Ribosomal_L12 (PF00542)	K02935
	4	symbB.v1.2.000698	3	1	3	symbB1.EST_k37c20_24144	40	Ribosomal_L12 (PF00542)	
rpl13		symbB.v1.2.021894	6	11	6	symbB1.EST_k37c20_11014	1900	Ribosomal_L13 (PF00572)	K02871
rpl14		symbB.v1.2.031130	4	3	1	symbB1.EST_k37c20_26909	3574	Ribosomal_L14 (PF00238)	K02874
rpl16		symbB.v1.2.034891	9	4	3	symbB1.EST_k37c20_186	4584	Ribosomal_L16 (PF00252)	K02878
rpl16		symbB.v1.2.026617	8	7	2	symbB1.EST_k37c20_61152	2674	Ribosomal_L16 (PF00252)	K02878
rpl20	1	symbB.v1.2.028173	3	4	0	symbB1.EST_k37c20_51249	2961	Ribosomal_L20 (PF00453)	K02887
	2	symbB.v1.2.034534	12	6	4	symbB1.EST_k37c20_4660, symbB1.EST_k37c20_5061	4475	Ribosomal_L20 (PF00453)	K02887
rpl21	1	symbB.v1.2.017666	8	10	2	symbB1.EST_k37c20_46407	1380	Ribosomal_L21p (PF00829)	K02888/0
	2	symbB.v1.2.017667	7	8	2	symbB1.EST_k37c20_46407	1380	Ribosomal_L21p (PF00829)	
rpl22		symbB.v1.2.026601	0	0	0	symbB1.EST_k37c20_59873	2672	Ribosomal_L22 (PF00237)	K02890
rpl23		symbB.v1.2.021021	8	4	3	symbB1.EST_k37c20_48685	1795	Ribosomal_L23 (PF00276)	
rpl24		symbB.v1.2.019792	13	6	1	symbB1.EST_k37c20_2539, symbB1.EST_k37c20_2538	1637	(PF00467)	K02895
rpl27		symbB.v1.2.022514	7	5	3	symbB1.EST_k37c20_38736	2003	Ribosomal_L27 (PF01016)	K02899
rpl31		symbB.v1.2.011799	3	9	1	symbB1.EST_k37c20_47182	773	Ribosomal_L31 (PF01197)	
rpl36		symbB.v1.2.012093	8	5	1	symbB1.EST_k37c20_43895	821	Ribosomal_L36 (PF00444)	
rps2		symbB.v1.2.016189	59	40	13	symbB1.EST_k37c20_30752, symbB1.EST_k37c20_8756	1223	Ribosomal_S2 (PF00318)	K02967
rps4		symbB.v1.2.009410	4	4	1	symbB1.EST_k37c20_3181, symbB1.EST_k37c20_3182	557	Ribosomal_S4 (PF00163)	K02986
rps9		symbB.v1.2.016652	6	6	4	symbB1.EST_k37c20_38309	1274	Ribosomal_S9 (PF00380)	K02996
rps12	1	symbB.v1.2.004458	3	6	2	symbB1.EST_k37c20_51368	2893	Ribosomal_S12 (PF00164)	K02950
	2	symbB.v1.2.027863	10	5	5	symbB1.EST_k37c20_6063	249	Ribosomal_S12 (PF00164)	K02950

rps16		symbB.v1.2.013041	7	10	2	symbB1.EST_k37c20_12315	915	Ribosomal_S16 (PF00886)	K02959
rps18		symbB.v1.2.019232	20	7	8	symbB1.EST_k37c20_80	1567	Ribosomal_S18 (PF01084)	
rps19		symbB1.v1.2.026125	0	0	0	symbB1.EST_k37c20_62582	2587	Ribosomal_S19 (PF00203)	K02965
**Protein quality control**									
clpC	1	symbB.v1.2.014468	8	8	4	symbB1.EST_k37c20_4653	1055	Clp_N (PF02861)	K03696
	2	symbB.v1.2.028955	22	14	8	symbB1.EST_k37c20_381	3121	Clp_N (PF02861)	K03696
clpP	1	symbB.v1.2.025383	7	10	1	symbB1.EST_k37c20_35751	2458	CLP_protease (PF00574)	K01358
	2	symbB.v1.2.028528	16	17	8	symbB1.EST_k37c20_43359	3032	CLP_protease (PF00574)	K01358
	3	symbB.v1.2.008467	14	7	6	symbB1.EST_k37c20_25146	474	CLP_protease (PF00574)	K01358
dnaK	1	symbB.v1.2.026933	8	10	3	symbB1.EST_k37c20_1571, symbB1.EST_k37c20_1570	2698	HSP70 (PF00012)	K04043
	2	symbB.v1.2.026931	6	6	1	symbB1.EST_k37c20_1569	2698	HSP70 (PF00012)	
	3	symbB.v1.2.026932	5	5	2	symbB1.EST_k37c20_1568	2698	HSP70 (PF00012)	
	4	symbB.v1.2.041606	6	5	1	symbB1.EST_k37c20_1568	1089	HSP70 (PF00012)	
	5	symbB.v1.2.014797	7	9	4	symbB1.EST_k37c20_16510	9398	HSP70 (PF00012)	
ftsH	1	symbB.v1.2.009970	21	12	12	symbB1.EST_k37c20_11246, symbB1.EST_k37c20_12744	623	AAA (PF00004)	K03798
	2	symbB.v1.2.026811	20	5	1	symbB1.EST_k37c20_11246	2711	AAA (PF00004)	K03798
	3	symbB.v1.2.022940	35	19	5	symbB1.EST_k37c20_12744	2066	AAA (PF00004)	K03798
	4	symbB.v1.2.028451	19	17	7	symbB1.EST_k37c20_11246	3018	AAA (PF00004)	K03798
groEL	1	symbB.v1.2.034222	21	12	7	symbB1.EST_k37c20_3046, symbB1.EST_k37c20_3045	4383	Cpn60_TCP1 (PF00118)	K04077
	2	symbB.v1.2.016853	22	14	5	symbB1.EST_k37c20_7154	1289	Cpn60_TCP1 (PF00118)	K04077
	3	symbB.v1.2.023783	30	19	8	symbB1.EST_k37c20_21115	2205	Cpn60_TCP1 (PF00118)	K04077
	4	symbB.v1.2.013239	24	25	4	symbB1.EST_k37c20_10912	891	Cpn60_TCP1 (PF00118)	
**Unknown**									
ycf3		symbB.v1.2.008172	13	9	5	symbB1.EST_k37c20_8962, symbB1.EST_k37c20_16423	512	TPR_11 (PF13414)	
ycf4		symbB.v1.2.027661	7	5	2	symbB1.comp5277_c0_seq1	2855	Ycf4 (PF02392)	
ycf19		symbB.v1.2.026242	7	7	1	symbB1.EST_k37c20_12378	2606	YGGT (PF02325)	K02221
ycf53		symbB.v1.2.006785	6	5	1	symbB1.EST_k37c20_13544	396	GUN4 (PF05419)	
ycf62/ mesJ		symbB.v1.2.012612	8	1	4	symbB1.EST_k37c20_43831	874	ATP_bind_3 (PF01171)	K04075
**rRNAs**									
16S		JX094331-2				symbB1.comp6517_c0_seq1, mf105_rep_c30787	minicircle		
23S		JX094333-5				symbB1.comp210_c1_seq10, mf105_rep_c958	minicircle		

Note: transcriptome ID, mf105_rep are transcriptomes independently generated for Roche 454 sequencer from Medina lab (Bayer et al. 2012).

Table S2. List of all amino-acid substitution found in plastid-encoded genes of *Symbiodinium minitum*.

	total	psbA	psbB	psbC	psbD	psbE	psbI	petB	petD	psaA	psaB	atpA	atpB
I→V	62	1	5	6	3	2	0	3	3	12	15	4	8
T→A	40	0	3	5	0	0	0	0	1	8	18	1	4
F→L	6	0	0	0	0	0	0	0	0	2	3	0	1
S→G	14	0	3	2	0	0	0	1	1	2	4	0	1
L→F	11	0	2	1	0	0	0	2	1	2	2	1	0
I→M	10	1	0	0	0	0	0	0	1	3	2	3	0
V→I	11	0	1	0	0	1	0	1	1	3	2	1	1
A→P	11	0	0	1	1	1	0	2	3	1	1	0	1
N→S	7	0	1	0	0	0	0	0	0	2	2	1	1
T→I	9	0	1	0	0	0	0	0	0	4	2	1	1
R→G	9	0	0	1	0	0	0	0	0	4	0	2	2
I→T	8	0	1	0	0	0	0	1	0	3	2	1	0
K→E	8	0	0	0	0	0	0	1	1	4	0	2	0
N→D	8	0	0	0	0	0	0	0	0	0	2	3	3
D→A	4	0	0	0	0	0	0	0	0	1	0	2	1
H→Y	5	0	0	0	0	0	0	0	0	1	3	0	1
I→A	8	0	1	0	0	0	0	0	0	3	2	1	1
L→S	5	0	1	0	0	0	0	1	0	1	0	0	2
M→V	7	0	2	0	0	1	0	0	1	1	0	2	0
S→F	7	0	1	1	0	0	0	1	0	3	1	0	0
T→V	5	0	0	1	0	0	0	0	0	2	0	1	1
F→S	5	0	0	0	0	0	1	1	0	0	0	2	1
G→A	3	0	0	0	0	0	0	3	0	0	0	0	0
Y→C	5	0	0	0	0	0	0	0	0	2	0	2	1
C→S	5	0	0	0	0	0	0	0	3	0	0	1	1
S→P	5	0	1	0	0	0	0	0	0	1	0	0	3
S→T	5	1	0	1	0	0	0	0	0	0	3	0	0
V→A	5	0	0	1	1	0	0	0	2	1	0	0	0
P→L	4	0	0	0	0	0	0	0	1	0	2	0	1
P→S	4	0	0	0	0	0	0	0	0	2	1	0	1
Y→S	3	0	0	1	0	0	0	0	0	1	1	0	0
A→T	3	0	0	0	0	0	1	0	0	0	2	0	0
E→G	2	0	0	0	0	0	0	0	1	0	0	0	1
H→R	3	0	0	0	0	0	0	0	0	0	2	0	1
K→R	3	0	1	0	0	0	0	0	0	0	1	1	0
N→G	2	0	0	0	0	0	0	0	0	0	1	0	1
S→L	3	0	0	0	0	0	1	0	0	2	0	0	0
T→M	2	0	0	0	0	0	0	0	0	0	1	1	0
A→V	2	0	0	0	0	1	0	0	0	1	0	0	0
C→R	2	0	1	0	0	0	0	0	1	0	0	0	0
D→S	2	0	0	0	0	0	0	0	0	2	0	0	0
G→R	1	0	0	0	0	0	0	1	0	0	0	0	0
G→S	2	0	1	0	0	0	0	0	0	0	1	0	0
H→C	2	0	0	0	0	0	0	0	0	1	0	1	0
I→L	2	0	0	0	0	0	0	0	0	2	0	0	0
K→A	2	0	0	0	0	0	0	0	1	1	0	0	0
K→G	2	0	0	0	0	0	0	0	0	1	0	0	1
K→N	2	0	0	0	0	2	0	0	0	0	0	0	0
L→P	2	0	0	0	1	0	0	0	1	0	0	0	0
Q→R	2	0	1	0	0	0	0	0	0	0	0	1	0
S→A	2	0	0	1	1	0	0	0	0	0	0	0	0
S→N	2	0	0	0	0	0	0	0	0	0	0	1	1
V→M	2	0	0	0	0	0	0	0	1	0	0	1	0
Y→H	1	0	0	0	0	0	0	1	0	0	0	0	0
*→W	1	0	0	0	0	0	0	0	1	0	0	0	0
C→P	1	0	0	0	0	0	0	0	1	0	0	0	0
D→A	1	0	0	0	0	0	0	0	0	1	0	0	0
D→H	1	0	0	0	0	0	0	0	0	1	0	0	0
D→G	1	0	0	0	0	0	0	0	0	0	1	0	0
F→P	1	0	0	0	0	0	0	1	0	0	0	0	0
K→T	1	0	0	0	0	0	0	0	0	0	0	0	1
L→R	0	0	0	0	0	0	0	0	0	0	0	0	0
M→A	1	0	0	0	0	0	0	0	0	1	0	0	0
M→T	1	0	0	0	0	0	0	0	0	1	0	0	0
N→V	1	0	0	0	0	0	0	0	0	0	1	0	0
R→E	1	0	0	0	0	0	0	0	0	1	0	0	0
R→T	1	0	0	0	0	0	0	1	0	0	0	0	0
S→D	1	0	1	0	0	0	0	0	0	0	0	0	0
V→L	1	0	0	0	0	0	0	0	1	0	0	0	0
V→T	1	0	0	0	0	0	0	0	1	0	0	0	0
W→R	1	0	0	0	0	0	0	0	0	0	0	0	1
*→S	1	0	0	0	0	0	0	1	0	0	0	0	0
	364												

Table S3. Comparison of RNA editing types and frequency of plastid-encoded genes among available dinoflagellates.

Gene	Dinoflagellates										
	Smi		Htr		Cho		Kve		Lpo		shared edit sites*
	edit types	no. edits	edit types	no. edits	edit types	no. edits	edit types	no. edits	edit types	no. edits	
psaA	A/G, G/A<br>C/U, U/C<br>G/C, A/C	100/2022	A/G<br>U/C<br>A/U	4//2196	A/G, G/A<br>C/U, U/C<br>G/C, A/C	104/1329	A/G, G/A<br>U/C	34/1227	nd		Smi&Htr: 1/670<br>Smi&Cho: 20/443<br>Smi&Kve: 5/380
psbB	A/G, G/A<br>C/U, U/C	30/1500	none	0	A/G, G/A<br>C/U, U/C<br>G/C, A/C	86/1560	nd		nd		Smi&Cho: 10/438
psbE	A/G, C/U<br>G/C, A/C<br>U/G	9/234	A/G<br>G/C	2/231	A/G, G/A<br>C/U, U/C<br>G/C	13/221	nd		nd		Smi&Cho: 2/70<br>Smi&Htr: 0
psbC	A/G, G/A<br>C/U, U/C<br>G/C, A/C	25/1359	A/G<br>G/C	4/1380	nd		A/G, G/A<br>C/U, U/C	25/904	nd		Smi&Kve: 4/307<br>Smi&Htr: 0
petD	A/G, G/A<br>C/U, U/C<br>G/C, A/C	33/477	A/G, U/C<br>G/C	5/474	nd		A/G, G/A<br>U/C	6/174	nd		Smi&Kve: 2/45<br>Smi&Htr: 1/158
petB	A/G, G/A<br>C/U, U/C<br>G/C	23/657	U/C	1/657	nd		nd		A/G, G/A<br>C/U, U/C	15/657	Smi&Gpo: 3/219<br>Smi&Htr: 0
psbA	A/G<br>G/C	3/1029	none	0	nd		nd		A/G, C/U<br>G/C	10/1035	Smi&Gpo: 2/342
psbD	A/G, C/U<br>G/C, U/G	8/1074	U/C	2/1071	nd		nd		nd		Smi&Htr: 0
atpA	A/G, G/A<br>C/U, U/C<br>G/C	43/1434	A/G, U/C<br>A/C	6/1437	nd		nd		nd		Smi&Htr: 4/478
16S	A/G, C/U	22/794	A/G, G/A<br>U/A	39/1514	A/G, C/U<br>U/C, U/G	35/976	nd		A/G, G/A<br>C/U, U/G	19/1159	
23S	A/G, C/U<br>G/C, G/U	36/1138	A/G, G/A<br>U/A, A/U	22/675	nd		nd		nd		

Smi, *Symbiodinium minitum;* Htr, *Heterocapsa triquetra*; Cho, *Ceratium horridum;* Kve, *Karlodinium veneficum;* Lpo, *Lingulodinium polyedra*

Note: 16S rRNA of dinoflagellates are quite diverged, difficult to locate comparable edited sites.
Comparison of edited sites between Smi and Htr was from the full coding sequence.
* Number of shared edited site that cause amino acid substitution in a comparable region
nd=data was not determined

TableS4. Number of editing site found on predicted secondary structure of plastid-encoded genes of *Symbiodinium minutum*.

Gene	Structures			Unpredicted sites	Total editied sites
	helix	sheet	others		
psbA	2	0	1	0	3
psbB	20	1	7	0	28
psbC	17	0	4	1	22
psbD	2	0	3	2	7
psbE	4	0	4	0	8
petB	14	0	8	0	22
petD	20	0	8	0	28
psaA	50	2	32	0	84
psaB	47	2	29	0	78
atpA	12	6	15	4	37
atpB	3	4	11	26	44
sum	191	15	122	33	361

Table S5. Changes of molecular weight and GRAVY by RNA editing of *Symbiodinium minitum* plastid-encoded genes.

Gene	MW before editing	MW after editing	MW change (%)	Gravy before editing	Gravy after editing	Gravy change (%)
psbA	37628.4	37646.4	0.05	0.533	0.525	-1.50
psbB	56017.5	55927.3	-0.16	0.25	0.243	-2.80
psbC	50455	50073.6	-0.76	0.224	0.27	20.54
psbD	40162.5	40086.4	-0.19	0.454	0.427	-5.95
psbE	8774	8753.9	-0.23	0.27	0.294	8.89
psbI	3716.3	3712.3	-0.11	1.097	1.054	-3.92
*petB**	24039.5	24074.4	0.15	0.813	0.729	-10.33
*petD**	17112.9	16985.4	-0.75	1.188	0.975	-17.93
psaA	75986.9	74723.3	-1.66	0.266	0.397	49.25
psaB	78679.1	77894.5	-1.00	0.229	0.33	44.10
atpA	51625.9	51052.1	-1.11	0.452	0.486	7.52
atpB	73594.7	72688.6	-1.23	0.109	0.148	35.78

*stop codon gives no MW and GRAVY score
